# Supplementary material for: Creating and validating the Fine-Grained Question Subjectivity Dataset (FQSD): A new benchmark for enhanced automatic subjective question answering systems
Source: PLoS One. 2024 May 23;19(5):e0301696. doi: 10.1371/journal.pone.0301696 (PMC11115256; doi:10.1371/journal.pone.0301696)
Supplement: Attachment — Submitted filename: Response to Reviewers.docx [file pone.0301696.s002.docx]

| **Date:** | Feb 27 2024 11:44AM |
| --- | --- |
| **To:** | "Afsaneh Fatemi" a_fatemi@eng.ui.ac.ir |
| **From:** | "PLOS ONE" plosone@plos.org |
| **Subject:** | PLOS ONE: Edits requested on your submission PONE-D-23-33628R1 |

=============================================

IMPORTANT: PLEASE DO NOT REPLY TO THIS EMAIL

If you are unable to complete any points that are requested in this email, please explain why in the 'Enter Comments' tab of the online submission form prior to re-submitting your manuscript. This will let us quickly assess your response and send your manuscript to an Academic Editor as soon as possible.

=============================================

PONE-D-23-33628R1
Creating and Validating the Fine-Grained Question Subjectivity Dataset (FSQD): a new benchmark for enhanced Automatic Subjective Question Answering systems
Dr. Afsaneh Fatemi

Dear Dr. Fatemi,

We've checked your submission and before we can proceed, we need you to address the following issues:

1. Please include a legend for figure 1.

We've returned your manuscript to your account. Please resolve these issues and resubmit your manuscript within 21 days. If you need more time, please email the journal office at plosone@plos.org. We are happy to grant extensions of up to one month past this due date. If we do not hear from you within 21 days, we will withdraw your manuscript.

Please log on to PLOS Editorial Manager at https://www.editorialmanager.com/pone/ to access your manuscript. You will find your manuscript in the 'Submissions Sent Back to Author' link under the New Submissions menu. Be sure to remove your previous manuscript file if you are uploading a new file in response to these requests. After you've made the changes requested above, please be sure to view and approve the revised PDF after rebuilding the PDF to complete the resubmission process.

We are requesting these changes to comply with the PLOS ONE submission guidelines (https://journals.plos.org/plosone/s/submission-guidelines). Please note that we won't send your manuscript for review until you have resolved the above requests.

Thank you for submitting your work to PLOS ONE and supporting our mission of Open Science.


Kind regards,

Oriel Jerome Delas Alas Vida
PLOS ONE

__________________________________________________
In compliance with data protection regulations, you may request that we remove your personal registration details at any time. (Use the following URL: https://www.editorialmanager.com/pone/login.asp?a=r). Please contact the publication office if you have any questions.

**Response to Editor's Invitation for Manuscript Revision**

Dear Editor,

Thank you for your letter and the opportunity to revise our manuscript titled. We appreciate the effort that the editorial team and reviewers put into reviewing our work, and we are committed to complying with PLOS ONE's submission guidelines.

In response to your request, we have included a detailed legend for Figure 1. The legend aims to provide a comprehensive understanding of the figure, ensuring that it can be interpreted accurately both within the context of our manuscript and as a standalone image.

We have carefully reviewed the entire manuscript to ensure that all other aspects comply with the submission guidelines and believe that the manuscript is now ready for further review. We have resubmitted the manuscript through the journal's submission system within the stipulated 21-day period.

We are grateful for the chance to improve our manuscript and contribute to the mission of Open Science. Please do not hesitate to contact us if you need further information or clarification regarding the revisions.

Thank you once again for your consideration and support.

Kind regards,

Dr. Afsaneh Fatemi

| **Date:** | Jan 11 2024 10:18AM |
| --- | --- |
| **To:** | "Afsaneh Fatemi" a_fatemi@eng.ui.ac.ir |
| **From:** | "PLOS ONE" plosone@plos.org |
| **Subject:** | PLOS ONE Decision: Revision required [PONE-D-23-33628] |

PONE-D-23-33628

Creating and Validating the Fine-Grained Question Subjectivity Dataset (FSQD): a new benchmark for enhanced Automatic Subjective Question Answering systems

PLOS ONE

Dear Dr. Fatemi,

Thank you for submitting your manuscript to PLOS ONE. After careful consideration, we feel that it has merit but does not fully meet PLOS ONE’s publication criteria as it currently stands. Therefore, we invite you to submit a revised version of the manuscript that addresses the points raised during the review process.

We appreciate the effort and expertise you have brought to your work; however, several areas have been identified that need significant improvement. Please find the detailed and actionable comments below. The comments from point three onwards are of particular importance. These suggestions are aimed at enhancing the clarity, depth, and overall quality of your manuscript. I concur with the reviewer comments and believe that addressing these points will substantially strengthen your paper. 

Please submit your revised manuscript by Feb 25 2024 11:59PM. If you will need more time than this to complete your revisions, please reply to this message or contact the journal office at [plosone@plos.org](mailto:plosone@plos.org). When you're ready to submit your revision, log on to https://www.editorialmanager.com/pone/ and select the 'Submissions Needing Revision' folder to locate your manuscript file.

Please include the following items when submitting your revised manuscript:

- A rebuttal letter that responds to each point raised by the academic editor and reviewer(s). You should upload this letter as a separate file labeled 'Response to Reviewers'.
- A marked-up copy of your manuscript that highlights changes made to the original version. You should upload this as a separate file labeled 'Revised Manuscript with Track Changes'.
- An unmarked version of your revised paper without tracked changes. You should upload this as a separate file labeled 'Manuscript'.

If you would like to make changes to your financial disclosure, please include your updated statement in your cover letter. Guidelines for resubmitting your figure files are available below the reviewer comments at the end of this letter.

If applicable, we recommend that you deposit your laboratory protocols in protocols.io to enhance the reproducibility of your results. Protocols.io assigns your protocol its own identifier (DOI) so that it can be cited independently in the future. For instructions see: <https://journals.plos.org/plosone/s/submission-guidelines#loc-laboratory-protocols>. Additionally, PLOS ONE offers an option for publishing peer-reviewed Lab Protocol articles, which describe protocols hosted on protocols.io. Read more information on sharing protocols at <https://plos.org/protocols?utm_medium=editorial-email&utm_source=authorletters&utm_campaign=protocols>.

We look forward to receiving your revised manuscript.

Kind regards,

John Blake, PhD
Academic Editor
PLOS ONE

**Response to Editor's Invitation for Manuscript Revision**

Dear Editor,

Dear Editor,

Thank you for considering our manuscript and for providing us with the opportunity to revise and resubmit it to PLOS ONE. We are grateful for the valuable feedback received from you and the reviewers. We believe that addressing these comments will greatly enhance the clarity, depth, and overall quality of our work.

We have carefully reviewed each point raised and have undertaken significant revisions to our manuscript to address the concerns highlighted. In particular, we have paid close attention to the comments from point three onwards, as per your recommendation.

In accordance with the submission guidelines, upon resubmitting our revised manuscript by the specified deadline, we will provide the following items:

1. **Rebuttal Letter:** A detailed letter responding to each point raised by the academic editor and reviewers. This document will be uploaded as a separate file labeled 'Response to Reviewers'.

2. **Marked-Up Manuscript:** A copy of our manuscript with track changes, highlighting the revisions made. This document will be uploaded as a separate file labeled 'Revised Manuscript with Track Changes'.

3. **Unmarked Manuscript:** An unmarked version of the revised paper without tracked changes. This document will be uploaded as a separate file labeled 'Manuscript'.

Additionally, we will review our financial disclosure statement and include any necessary updates in our cover letter.

Regarding the suggestion to deposit our laboratory protocols in protocols.io, we [include your decision on this suggestion, e.g., "appreciate this recommendation and will be depositing our protocols accordingly" or "will consider this for future research, as our current study did not involve laboratory protocols"].

We are committed to enhancing the reproducibility and transparency of our research and appreciate the guidance provided by PLOS ONE in this process.

We look forward to the opportunity to contribute to the PLOS ONE community and thank you again for your valuable feedback. Please do not hesitate to contact us if further information or clarification is required in the meantime.

Kind regards,

Dr. Afsaneh Fatemi

**Opening Response to the Editor:**

Dear Editor,

Thank you for overseeing the review process of our manuscript and for conveying the valuable feedback from Reviewer #1. We are grateful for the opportunity to refine our work based on these insightful comments. The suggestions provided have been instrumental in guiding our revisions, and we have addressed each point in detail to enhance the quality and clarity of our manuscript in the field of Question Answering (QA) in Natural Language Processing (NLP).

Journal requirements:

When submitting your revision, we need you to address these additional requirements.

1. Please ensure that your manuscript meets PLOS ONE's style requirements, including those for file naming. The PLOS ONE style templates can be found at

https://journals.plos.org/plosone/s/file?id=wjVg/PLOSOne_formatting_sample_main_body.pdf and

<https://journals.plos.org/plosone/s/file?id=ba62/PLOSOne_formatting_sample_title_authors_affiliations.pdf>

**Response to Editor's Comment 1: Compliance with PLOS ONE Style Requirements**

Dear Editor,

Thank you for directing us to PLOS ONE's style requirements. We appreciate your guidance in ensuring that our manuscript adheres to the journal's formatting standards.

In response to your comment, we have thoroughly reviewed the PLOS ONE style templates and made the necessary adjustments to our manuscript to ensure full compliance. This includes:

**1. Manuscript Formatting:** We have revised the manuscript format according to the guidelines provided in the PLOS ONE formatting sample for the main body. This includes adjustments to the text layout, font sizes, heading styles, and other formatting elements as per the template.

**2. Title, Authors, and Affiliations:** We have also reformatted the title, authors, and affiliations section of our manuscript in line with the PLOS ONE formatting sample for title, authors, and affiliations. We have ensured that all necessary information is presented clearly and in the correct order.

**3. File Naming Convention:** Additionally, we have revised the file naming of our manuscript and supporting documents to align with PLOS ONE's requirements. We have ensured that all files associated with our submission are appropriately named for ease of identification and organization.

We believe these revisions have brought our manuscript fully in line with PLOS ONE's style guidelines. We are committed to maintaining the highest standards of presentation and are grateful for your assistance in this process.

Please find the revised manuscript attached for your review. We look forward to any further feedback or instructions you might have.

2. Note from Emily Chenette, Editor in Chief of PLOS ONE, and Iain Hrynaszkiewicz, Director of Open Research Solutions at PLOS: Did you know that depositing data in a repository is associated with up to a 25% citation advantage (https://doi.org/10.1371/journal.pone.0230416)? If you’ve not already done so, consider depositing your raw data in a repository to ensure your work is read, appreciated and cited by the largest possible audience. You’ll also earn an Accessible Data icon on your published paper if you deposit your data in any participating repository (<https://plos.org/open-science/open-data/#accessible-data>).

**Response to Note from Emily Chenette and Iain Hrynaszkiewicz**

Dear Editor,

Thank you for bringing to our attention the potential citation advantage associated with depositing data in a repository, as highlighted in your recent publication (https://doi.org/10.1371/journal.pone.0230416). We greatly appreciate this valuable insight and the opportunity to enhance the impact and accessibility of our research.

In response to your suggestion:

**1. Data Repository Deposition:**

We are pleased to announce the deposition of our raw data in a GitHub repository, adhering to PLOS's open data policies. This initiative underscores our commitment to open science by ensuring our data is accessible to the research community. The dataset can be found at https://github.com/mahsamb/FSQD, and the code related to our figures and model is at: https://github.com/mahsamb/FSQD/blob/main/Figures.ipynb.

**2. Earning the Accessible Data Icon:** We are also keen to contribute to the Open Science movement and earn the Accessible Data icon on our published paper. We understand that depositing our data in a participating repository will facilitate this and are committed to following through with this step.

We believe that by making our data openly accessible, we can help ensure that our work reaches the widest possible audience and fosters further research and collaboration in our field.

Thank you for your guidance and support in making our research more accessible and impactful. We look forward to being a part of PLOS's open science initiatives.

3. In your Methods section, please include additional information about your dataset and ensure that you have included a statement specifying whether the collection and analysis method complied with the terms and conditions for the source of the data.

**Response to Editor's Comment on Dataset Modification and Creation**

Dear Editor,

We appreciate the constructive feedback and the opportunity to enhance the clarity and quality of our work. In particular, we have taken careful note of the request for additional information about our dataset and the compliance of our collection and analysis methods with the terms and conditions for the source of the data.

In response to this, we have added a new section to our “Methodology” section, titled "3.2.1 Dataset Preparation and Specifications," to explicitly address these concerns. Below, we outline the key components of this new section and how it addresses the reviewer's comments:

In this subsection, we detail the rigorous processes behind the dataset's development, including:

- **Dataset Inspiration and Collection:** We describe our initial dataset's inspiration from user-generated content on platforms like Quora and Amazon, focusing on the smartphone domain and adhering to specific research criteria.
- **Adaptation and Creation of the FQSD Dataset:** To address the scarcity of directly applicable questions, we adapted existing content and created new questions, ensuring a rich diversity of data. This involved modifying sentences, adjusting content, and creating fictional but plausible questions to broaden the dataset's scope.
- **Compliance and Ethical Considerations:** We have explicitly stated our strict adherence to the terms and conditions of the source platforms. Our processes for data collection, modification, and creation were conducted with the utmost respect for privacy, confidentiality, and intellectual property rights. This includes of our compliance efforts and the measures taken to ensure the exclusion of personally identifiable information.

By incorporating "3.2.1 Dataset Preparation and Specifications," into our methodology, we aim to provide a clear and thorough explanation of our dataset preparation, ethical considerations, and compliance with source data terms and conditions. We believe this addition addresses the reviewer's concerns and enhances the manuscript's transparency and rigor.

We are grateful for the opportunity to clarify these aspects of our work and thank the reviewers for their valuable insights. We look forward to any further feedback.

**To honor the editor's input and ensure the thoroughness of our response, we also include this addition here in our response letter:**

**3.2. Data Quality and Integrity**

Ensuring the standards of data quality and integrity is foundational to the development and utility of the FQSD dataset. This segment elaborates on the processes employed to cultivate a dataset that meets the demands of NLP research. Through comprehensive dataset preparation, the integration of unique features, a carefully designed annotation process, and stringent measures to gauge inter-annotator agreement, we establish an approach that prioritizes precision, reliability, and ethical considerations in dataset creation.

**3.2.1. Dataset Preparation and Specifications**

In this section, an exhaustive and detailed account of the rigorous preparation and underlying specifications that form the cornerstone of this dataset's development is provided. This includes an in-depth exploration of the methods employed, the criteria applied in the selection and adaptation of data, and the strategic considerations that guided the compilation process to ensure the dataset's integrity and relevance to its intended research applications.

- **Dataset Inspiration and Collection:** Our initial dataset drew inspiration from user-generated content on question-answering platforms such as Quora and Amazon, specifically in the smartphone domain. However, sourcing questions that met the specific criteria proved challenging. The criteria necessitated the explicit mention of compared entities' names, clarity, and specificity in phrasing, and alignment with our predefined categories.
- **Adaptation and Creation of the FQSD Dataset:** There is limited availability of questions directly fitting our research needs. To fill the gaps, we adapted existing content and created new questions, ensuring a rich variety of data. This included:
  - **Modify Sentences:** Transforming existing sentences into questions suitable for our study.
  - **Content Adjustment:** Adding, removing, or altering sections of the questions to enhance specificity and relevance.
  - **Creating Fictional Data:** Supplementing the dataset with fictional but plausible questions to cover a broader range of scenarios and inquiries.
- **Validation and Approval Process:** The altered and newly created questions were subjected to a rigorous validation process, overseen by expert commentators knowledgeable in writing and conceptual aspects within the smartphone domain. Their approval ensured the dataset's relevance and adherence to our research objectives.
- **Bias Mitigation Strategies in Dataset Compilation:** To ensure the FQSD dataset minimizes biases inherent in question selection and framing, several strategies tailored to question classification tasks were implemented. Firstly, the dataset was compiled from a wide range of online platforms beyond mainstream sites like Quora and Amazon, including niche forums and industry-specific Q&A sites. This approach ensures a variety of question types and topics, reducing the risk of selection bias towards popular or commonly discussed issues. Secondly, during the adaptation and creation of questions, we carefully balanced the dataset to include questions from different smartphone categories, reflecting a broad spectrum of user intents and information needs. This diversity helps in minimizing content bias, ensuring our dataset supports the development of classification models capable of understanding a wide range of question formats and types. Finally, to address annotator bias in question classification, annotators were provided with extensive guidelines designed to maintain consistency and neutrality in question interpretation and classification. Regular calibration sessions were held to align annotators’ understanding and ensure a uniform standard was applied across the dataset.
- **Compliance and Ethical Considerations:** Our data collection, modification, and creation processes were conducted in strict compliance with the terms and conditions of the source platforms. Ethical considerations were paramount, including the exclusion of any personally identifiable information and ensuring the privacy and confidentiality of individuals associated with the initial data sources.

4. Please note that PLOS ONE has specific guidelines on code sharing for submissions in which author-generated code underpins the findings in the manuscript. In these cases, all author-generated code must be made available without restrictions upon publication of the work. Please review our guidelines at https://journals.plos.org/plosone/s/materials-and-software-sharing#loc-sharing-code and ensure that your code is shared in a way that follows best practice and facilitates reproducibility and reuse.

To respond to the editor's note regarding PLOS ONE's guidelines on code sharing, you should acknowledge the importance of sharing your author-generated code and confirm your commitment to making it available in accordance with their guidelines. Here's a suggested format for your response:

**Response to Editor's Comment on Code Sharing Guidelines**

Dear Editor,

Thank you for bringing to our attention the specific guidelines of PLOS ONE regarding the sharing of author-generated code. We fully understand the importance of making our code available to ensure the reproducibility and reuse of our research findings.

In compliance with PLOS ONE's code sharing policy, as outlined at https://journals.plos.org/plosone/s/materials-and-software-sharing#loc-sharing-code, we have taken the following steps:

1. **Code and Data Availability and Repository:** We have uploaded our code and data to GitHub repository, which is a recognized and accessible platform for code sharing in the scientific community. The dataset can be found at https://github.com/mahsamb/FSQD, and the code related to our figures and model is at: <https://github.com/mahsamb/FSQD/blob/main/Figures.ipynb> and <https://github.com/mahsamb/FSQD/blob/main/RoBERTa_Model.ipynb> . The repository link will be provided in the manuscript to ensure easy access for readers. This includes detailed scripts for the generation of Figures, as well as the implementation of our FQSC model, ensuring full transparency and enabling reproducibility of our findings.
2. **Documentation and Licensing:** Our code is well-documented to facilitate its understanding and use by others. We have also included a clear license that outlines the terms under which our code can be used, ensuring compliance with PLOS ONE's guidelines for unrestricted access.
3. **Best Practices for Code Sharing:** We have reviewed and adhered to best practices in code sharing to enhance the reproducibility of our work. This includes providing detailed comments within the code, a comprehensive README file, and ensuring the code is well-structured and organized.

We believe that by making our code readily available and easily accessible, we are contributing to the openness and reproducibility of scientific research. We are committed to upholding the standards set by PLOS ONE and appreciate the journal's emphasis on transparent and open science practices.

Thank you for your guidance in this matter. We are ready to make any further adjustments as needed to meet the journal's requirements fully.

**To honor the editor's input and ensure the thoroughness of our response, we also include this addition here in our response letter:**

**6. Code and Data Availability**

To ensure the reproducibility of our findings and facilitate further research in this area, the dataset used in this study and the corresponding code have been made publicly available. The dataset can be accessed at https://github.com/mahsamb/FSQD, and the code repository is available at <https://github.com/mahsamb/FSQD/blob/main/Figures.ipynb>.

5. We note that your Data Availability Statement is currently as follows: [All relevant data are within the manuscript and its Supporting Information files.]

Please confirm at this time whether or not your submission contains all raw data required to replicate the results of your study. Authors must share the “minimal data set” for their submission. PLOS defines the minimal data set to consist of the data required to replicate all study findings reported in the article, as well as related metadata and methods (https://journals.plos.org/plosone/s/data-availability#loc-minimal-data-set-definition).

For example, authors should submit the following data:

- The values behind the means, standard deviations and other measures reported;

- The values used to build graphs;

- The points extracted from images for analysis.

Authors do not need to submit their entire data set if only a portion of the data was used in the reported study.

If your submission does not contain these data, please either upload them as Supporting Information files or deposit them to a stable, public repository and provide us with the relevant URLs, DOIs, or accession numbers. For a list of recommended repositories, please see https://journals.plos.org/plosone/s/recommended-repositories.

If there are ethical or legal restrictions on sharing a de-identified data set, please explain them in detail (e.g., data contain potentially sensitive information, data are owned by a third-party organization, etc.) and who has imposed them (e.g., an ethics committee). Please also provide contact information for a data access committee, ethics committee, or other institutional body to which data requests may be sent. If data are owned by a third party, please indicate how others may request data access.

example you provided:

________________________________________

**Response to Editor's Comment 3: Dataset Description and Compliance**

Dear Editor,

In line with the guidelines set forth by PLOS for data transparency and replicability, we have ensured that all raw data underpinning the findings of our study are comprehensively deposited and made publicly available. Our commitment to advancing open science practices is reflected in our decision to host the dataset on GitHub, a platform renowned for its accessibility and reliability within the scientific community. This repository not only contains the raw data necessary to replicate the results presented in our manuscript but also includes detailed metadata and methodological descriptions to facilitate thorough understanding and reuse. The dataset, alongside the relevant scripts and resources, is accessible via our dedicated GitHub repository at https://github.com/mahsamb/FSQD. We believe that this level of transparency and openness will foster further research and collaboration in our field. Thank you for guiding us to refine our manuscript. We eagerly await your further suggestions and feedback.

**To honor the editor's input and ensure the thoroughness of our response, we also include this addition here in our response letter:**

**6. Code and Data Availability**

To ensure the reproducibility of our findings and facilitate further research in this area, the dataset used in this study and the corresponding code have been made publicly available. The dataset can be accessed at https://github.com/mahsamb/FSQD, and the code repository is available at <https://github.com/mahsamb/FSQD/blob/main/Figures.ipynb>.

6. We note that Figures 3, 4, 5 and 6 in your submission contain copyrighted images. All PLOS content is published under the Creative Commons Attribution License (CC BY 4.0), which means that the manuscript, images, and Supporting Information files will be freely available online, and any third party is permitted to access, download, copy, distribute, and use these materials in any way, even commercially, with proper attribution. For more information, see our copyright guidelines: http://journals.plos.org/plosone/s/licenses-and-copyright.

We require you to either (1) present written permission from the copyright holder to publish these figures specifically under the CC BY 4.0 license, or (2) remove the figures from your submission:

1. You may seek permission from the original copyright holder of Figures 3, 4, 5 and 6 to publish the content specifically under the CC BY 4.0 license.

We recommend that you contact the original copyright holder with the Content Permission Form (http://journals.plos.org/plosone/s/file?id=7c09/content-permission-form.pdf) and the following text:

“I request permission for the open-access journal PLOS ONE to publish XXX under the Creative Commons Attribution License (CCAL) CC BY 4.0 (http://creativecommons.org/licenses/by/4.0/). Please be aware that this license allows unrestricted use and distribution, even commercially, by third parties. Please reply and provide explicit written permission to publish XXX under a CC BY license and complete the attached form.”

Please upload the completed Content Permission Form or other proof of granted permissions as an "Other" file with your submission.

In the figure caption of the copyrighted figure, please include the following text: “Reprinted from [ref] under a CC BY license, with permission from [name of publisher], original copyright [original copyright year].”

2. If you are unable to obtain permission from the original copyright holder to publish these figures under the CC BY 4.0 license or if the copyright holder’s requirements are incompatible with the CC BY 4.0 license, please either i) remove the figure or ii) supply a replacement figure that complies with the CC BY 4.0 license. Please check copyright information on all replacement figures and update the figure caption with source information. If applicable, please specify in the figure caption text when a figure is similar but not identical to the original image and is therefore for illustrative purposes only.

**Response to Editor Regarding Use of Word Clouds**

Dear Editor,

Thank you for your feedback regarding the word clouds depicted in Figures 3, 4, 5, and 6. In response to the concerns raised regarding the copyright status of Figures 3, 4, 5, and 6 in our submission, we have elected to remove the mentioned word cloud images from our manuscript. **To maintain the integrity and depth of our analysis, we have instead adopted an alternative exploration method, specifically utilizing TF-IDF (Term Frequency-Inverse Document Frequency) analysis.** This approach allows us to quantitatively evaluate the importance of words in our dataset while adhering to the Creative Commons Attribution License (CC BY 4.0) requirements set forth by PLOS. We believe this modification not only complies with copyright guidelines but also enriches our submission by providing a nuanced perspective on the textual data under investigation. The manuscript has been updated accordingly to reflect these changes, and we trust that this resolution addresses the copyright concerns satisfactorily. We appreciate the opportunity to clarify this aspect of our manuscript.

**To honor the editor's input and ensure the thoroughness of our response, we also include this addition here in our response letter:**

**Fig 1.** Top 30 TF-IDF Scores of Nouns and Noun Phrases in FQSD.

**Fig 2.** Top 20 TF-IDF Scores of Adverbs/Adjectives in FQSD Categorized by Subjectivity Comparison-Form Classes: a) CO b) CS c) SO d) SS

**Fig 3.** Histograms of TF-IDF Adverbs/Adjectives Scores in FQSD Categorized by Subjectivity Comparison-Form Classes: a) CO b) CS c) SO d) SS

**Conclusion Response to the Editor:**

In conclusion, we have thoroughly revised our manuscript, taking into account each of the constructive comments provided by Reviewer #1. These revisions have not only addressed the concerns raised but also added depth and clarity to our research. We believe that our manuscript now presents a more comprehensive and clear contribution to the field of NLP and QA.

**Opening Response to the Reviewers:**

Dear Reviewer,

Thank you for your insightful comments and the positive recognition of our work's contribution to the field of Question Answering (QA) in Natural Language Processing (NLP). We appreciate your detailed review and the opportunity to address the areas where you have suggested improvements. We are committed to enhancing the quality and clarity of our manuscript and have made revisions accordingly.

Reviewer's Responses to Questions

**Comments to the Author**

1. Is the manuscript technically sound, and do the data support the conclusions?

The manuscript must describe a technically sound piece of scientific research with data that supports the conclusions. Experiments must have been conducted rigorously, with appropriate controls, replication, and sample sizes. The conclusions must be drawn appropriately based on the data presented.

Reviewer #1: Yes

**Response to Reviewer #1's Comment on Technical Soundness and Data Support**

Dear Reviewer,

We are grateful for your evaluation of our manuscript as technically sound and for recognizing that our data supports the conclusions drawn. Your confirmation that our research adheres to rigorous scientific standards is greatly appreciated and reinforces the validity of our work.

2. Has the statistical analysis been performed appropriately and rigorously?

Reviewer #1: N/A

**Response to Reviewer #1's Comment on Statistical Analysis**

Dear Reviewer,

Thank you for your assessment regarding the statistical analysis portion of our manuscript. Following your insightful comment, we have rigorously reviewed and strengthened our methodologies to ensure their appropriateness and robustness, thereby enhancing the reliability of our findings. We hope these revisions adequately address and resolve your concerns.

3. Have the authors made all data underlying the findings in their manuscript fully available?

The [PLOS Data policy](http://www.plosone.org/static/policies.action#sharing) requires authors to make all data underlying the findings described in their manuscript fully available without restriction, with rare exception (please refer to the Data Availability Statement in the manuscript PDF file). The data should be provided as part of the manuscript or its supporting information, or deposited to a public repository. For example, in addition to summary statistics, the data points behind means, medians and variance measures should be available. If there are restrictions on publicly sharing data—e.g. participant privacy or use of data from a third party—those must be specified.

Reviewer #1: Yes

**Response to Reviewer #1's Comment on Data Availability**

Dear Reviewer,

We are thankful for your acknowledgment regarding the availability of all data underlying the findings in our manuscript. Your confirmation is greatly appreciated and aligns with our commitment to adhere to the PLOS Data policy.

4. Is the manuscript presented in an intelligible fashion and written in standard English?

PLOS ONE does not copyedit accepted manuscripts, so the language in submitted articles must be clear, correct, and unambiguous. Any typographical or grammatical errors should be corrected at revision, so please note any specific errors here.

Reviewer #1: Yes

**Response to Reviewer #1's Comment on Manuscript Presentation and Language Quality**

Dear Reviewer,

We are grateful for your affirmation that our manuscript is presented in an intelligible manner and is written in standard English. Your confirmation that our submission meets the language standards required by PLOS ONE is highly encouraging.

5. Review Comments to the Author

Please use the space provided to explain your answers to the questions above. You may also include additional comments for the author, including concerns about dual publication, research ethics, or publication ethics. (Please upload your review as an attachment if it exceeds 20,000 characters)

Reviewer #1: The paper under discussion is indeed quite intriguing and contributes significantly to the field of Question Answering (QA) Natural Language Processing (NLP). However, there are some areas where the authors can make improvements to enhance the overall quality and clarity of their work:

Dear Reviewer,

Thank you sincerely for your insightful and constructive feedback on our manuscript. Your comments are invaluable in guiding us to enhance the clarity and quality of our work in Question Answering (QA) and Natural Language Processing (NLP). In the following, we address each of your points in detail to ensure that our revisions align with your recommendations.

**1. Visualization Choice and Accessibility:**

a) The usage of word clouds in scientific works is not very common nowadays. It is advisable to replace them with more informative and visually accessible alternatives. Options such as Term Frequency-Inverse Document Frequency (TF-IDF) representations, t-Distributed Stochastic Neighbor Embedding (t-SNE), or topic modeling could provide richer insights.

Dear Reviewer,

Thank you for your valuable feedback regarding our use of word clouds and the suggestion to employ more contemporary visualization techniques. We appreciate your emphasis on informativeness of visual data representations.

**Alternative Visualization Techniques:** In response to your suggestion, we have explored incorporating Term Frequency-Inverse Document Frequency (TF-IDF) representation as an alternative to word clouds. This technique has allowed us to quantitatively evaluate and visually represent the significance of words within the FQSD dataset, offering a more nuanced understanding of key terms across different question categories. By implementing TF-IDF, we have identified and highlighted the most impactful nouns, noun phrases, adjectives, and adverbs that characterize each class, providing deep insights into the linguistic patterns and subjectivity within the dataset. This approach has not only enriched our analysis but also enhanced the clarity and depth of our findings, enabling us to draw more precise conclusions about the nature of discourse in question classification systems.

**To honor the reviewer's input and ensure the thoroughness of our response, we also include this addition here in our response letter:**

**4.1.1 TF-IDF's Exploration of Key Terms Within FQSD**

In this section, we leverage TF-IDF to visually dissect the FQSD, highlighting key terms in each class. TF-IDF, a statistical technique for determining the importance of a word in a document compared to a larger collection of documents, reveals significant and crucial terms, thus outlining unique language patterns within the various categories of the dataset. This method is instrumental in enhancing text analysis, allowing us to pinpoint and elevate the most telling words that define each category within FQSD.

Fig 1 presents the top 30 unigram, bigram, and trigram TF-IDF terms, emphasizing significant nouns and noun phrases within the FQSD dataset. Consistent with previous research [37-38], entities and their attributes tend to manifest as nouns or noun phrases. The graph reveals prominent entities such as 'Samsung Galaxy', 'iPhone', and 'Xiaomi', alongside key aspects like 'camera', 'quality', and 'display', indicating these are focal points in questions. To achieve this, we utilize the spaCy library for precise natural language processing (NLP) to preprocess text, specifically targeting the extraction of significant nouns and noun phrases. The methodology employs the TfidfVectorizer from scikit-learn to compute TF-IDF scores, followed by sorting these scores to unearth the most impactful terms.

**Fig 1. Top 30 TF-IDF Scores of Nouns and Noun Phrases in FQSD.**

Furthermore, grasping the significance of adjectives and adverbs is essential for discerning subjectivity/objectivity and comparative/non-comparative context in textual data. In this context, for visual analysis via TF-IDF, we deliberately targeted these parts of speech within four streamlined classes: Comparative Subjective (CS), Comparative Objective (CO), Single Subjective (SS), and Single Objective (SO). This focus is justified by the observed linguistic overlap among the ten initial classes, where related categories demonstrated homogeneous usage of descriptors, thus obscuring the boundaries essential for detailed examination. By focusing the analysis on CS, CO, SS, and SO, analytical clarity is maintained, avoiding the dilution of distinctiveness and ensuring a concise representation of the data. In Fig 2, the top 20 terms for each class illuminate the discourse nature within these categories. For instance, "more," "higher," and "faster" are prominent in CO, suggesting quantifiable comparisons. CS terms like "better" and "superior" reflect evaluative judgments in comparative assessments. The SO class includes terms such as "fast," "compatible," and "expandable," focusing on objective attributes, while SS features terms like "satisfied," "well," and "good," indicating subjective personal assessments. To achieve this, we utilize the spaCy library to preprocess text, specifically targeting the extraction of significant adjectives and adverbs. The methodology employs the TfidfVectorizer from scikit-learn, configured to analyze only unigrams, to compute TF-IDF scores. These scores are then sorted to unearth the most impactful terms, emphasizing the focused and nuanced use of vocabulary within the FQSD dataset. It provides insights into the linguistic nuances and the focused use of vocabulary within the FQSD dataset.

**Fig 1.** Top 30 TF-IDF Scores of Nouns and Noun Phrases in FQSD.

**Fig 2.** Top 20 TF-IDF Scores of Adverbs/Adjectives in FQSD Categorized by Subjectivity Comparison-Form Classes: a) CO b) CS c) SO d) SS

In addition, Fig 3 showcases histograms of the TF-IDF scores for unigram adjectives and adverbs across four distinct classes—Comparative Objective (CO), Comparative Subjective (CS), Single Objective (SO), and Single Subjective (SS). It visualizes the distribution of non-zero TF-IDF scores for adjectives and adverbs in the dataset, and generates histograms to illustrate the variance and concentration of TF-IDF scores within each category. This visualization serves to further validate our analytical methodology. A closer examination of the histograms for each category uncovers the unique linguistic patterns that highlight our findings:

- **Comparative Objective (CO):** The histogram indicates a pronounced peak at higher TF-IDF scores, reflecting a concentrated use of specific terms critical for objective comparisons. Terms like "more," "higher," and "faster" dominate, suggesting a narrow, targeted linguistic approach in CO questions.
- **Comparative Subjective (CS):** This class's histogram shows a broader distribution of TF-IDF scores, with a less sharp peak, indicating a diverse vocabulary used to express subjective comparisons. Terms such as "better" and "superior" are prevalent, highlighting the nuanced and varied language in CS questions.
- **Single Objective (SO) and Single Subjective (SS):** Both classes exhibit trends similar to their comparative counterparts, with SO showing a sharp peak and SS displaying a more even distribution of higher TF-IDF scores. The SO class focuses on specific objective attributes with terms like "fast," "compatible," and "expandable," while the SS class reveals a balanced use of language reflecting personal evaluations, highlighted by terms such as "satisfied," "well," and "good."

The distinct peaks at the higher end of the TF-IDF spectrum for CO and CS classes underscore the specificity of language in comparative questions, whereas the broader distribution in CS and the balanced distribution in SS suggest a richer vocabulary for subjective expressions and personal evaluations. These patterns validate the analytical approach, demonstrating that the four-class categorization effectively captures the linguistic essence of user queries in the dataset, showcasing clear lexical patterns that justify the segmented analysis for a nuanced understanding of language use within each category.

Classes: a) CO b) CS c) SO d) SS

**Fig 3.** Histograms of TF-IDF Adverbs/Adjectives Scores in FQSD Categorized by Subjectivity Comparison-Form Classes: a) CO b) CS c) SO d) SS

b) Moreover, it's important to ensure that the chosen visualizations are color-blind friendly to make the paper accessible to a wider audience.

Dear Reviewer,

Thank you for highlighting the critical aspect of accessibility in research dissemination, specifically the need for color-blind friendly visualizations in our manuscript. Your insight is invaluable in our endeavor to make our work inclusive and accessible to all members of the scientific community.

**Ensuring Accessibility:** We fully recognize the importance of making our paper accessible to a wider audience, including those with color vision deficiencies. Consequently, we have revised our visualizations to be color-blind friendly, employing a palette that is distinguishable in such scenarios. In this regard, figures 1-10 (of the revised manuscript) have been updated with color schemes optimized for color-blind readers, ensuring that our findings are comprehensible to everyone, regardless of their visual capabilities. This adjustment not only aligns with best practices in scientific communication but also reinforces our commitment to inclusivity in academic publishing.

**To honor the reviewer's input and ensure the thoroughness of our response, we also include this addition here in our response letter:**

**Fig 4.** Distribution of Total Question Count and Multi-Sentence Question Count across the FQSD, ConvEx-DS, Yu et al., 2012, and SubjQA datasets, showcasing the Size and Structural Analysis of each dataset

**Fig 5.** Distribution of the Total Word Count and Unique Word Count across the FQSD, ConvEx-DS, Yu et al., 2012, and SubjQA datasets, showcasing the Lexical Richness of each dataset.

**Fig 6.** Distribution of the Average Words per Sentence, Average Sentence Length, Average Word Length, and Average Syllables per Word across the FQSD, ConvEx-DS, Yu et al., 2012, and SubjQA datasets, showcasing the Linguistic Complexity of each dataset.

**Fig 7.** Visualizing the Average Parse Tree Depth across the FQSD, ConvEx-DS, Yu et al., 2012, and SubjQA datasets, showcasing the Syntactic Complexity of each dataset.

**Fig 8.** Visualizing the Mean Dependency Distance (MDD) across the FQSD, ConvEx-DS, Yu et al., 2012, and SubjQA datasets, showcasing the Dependency Analysis of each dataset.

**Fig 9.** Visualizing the Root Type-Token Ratio (RTTR) and the Corrected Type-Token Ratio (CTTR) across the FQSD, ConvEx-DS, Yu et al., 2012, and SubjQA datasets, showcasing the Lexical Diversity of each dataset.

**Fig 10.** Visualizing the Sparsity Degree and Total Question Count across the FQSD, ConvEx-DS, Yu et al., 2012, and SubjQA datasets, showcasing the Data Sparsity of each dataset.

**c)** Lastly, it's essential that the visualizations effectively convey the intended message.

Dear Reviewer,

Thank you for your valuable feedback regarding our use of word clouds and the suggestion to employ more contemporary visualization techniques. We appreciate your emphasis on both the informativeness and accessibility of visual data representations.

**Effectiveness of Visualizations:** We have carefully reviewed our visualizations to ensure that they effectively convey the intended message. Our goal is to provide clarity and enhance the reader's understanding of the key findings and patterns in our data. To this end:

- **Refined Captions:** We have refined the captions of our figures to be more informative, aiding in the interpretation and understanding of the visual content.
- **Revised Contextual Explanations:** We thoroughly revised the context related to our figures to ensure clarity and relevance.
- **Overhauled "4.1.2. Comparative Analysis" Section:** We almost completely reworked this crucial subsection of "4.1. Dataset Evaluation" to better explain the rationale behind our choice of metrics for evaluating the dataset depicted in the figures.
- **Ensured Alignment with Analytical Narrative:** We ensured that each visualization precisely complements the textual explanations, contributing to a holistic understanding of our research findings.
- **Released Code on GitHub:** We have released our code related to the figures in our manuscript on GitHub at: <https://github.com/mahsamb/FSQD/blob/main/Figures.ipynb>. In this Jupyter Notebook, we illustrate the libraries, functions, and visualization codes used in our study and provide clarifications for each. This ensures transparency and facilitates replication or further exploration by interested researchers.
- **Revised Visualizations for Figures 11 and 13 (in old manuscript version):** We updated the visualizations in figures 9 and 10 (revised version) to enhance clarity. Specifically, for Figure 10, we adopted a better metric for demonstrating the degree of sparsity, thereby providing a clearer and more accurate representation of our data's characteristics.

We hope that these revisions meet your expectations and significantly improve the quality of our manuscript. We are grateful for your guidance, which has helped us enhance the overall presentation and accessibility of our work.

**To honor the reviewer's input and ensure the thoroughness of our response, we also include this addition here in our response letter:**

**The revised version of the “Data Sparsity” section is as follows:**

**Data Sparsity**

In the pursuit of enhancing our understanding and analysis of datasets used for FQSC, we adopted a metric for assessing the linguistic diversity of each dataset: Sparsity Degree. This metric is pivotal in quantifying the extent of vocabulary diversity, which is essential for tasks requiring nuanced language interpretation, such as subjectivity classification. The Sparsity Degree is calculated using the formula [56]:

$$Sd=1- \frac{1}{n} \sum_{i=1}^{n} \frac{N_{i}}{\left| V \right|} (1)$$

Where *Sd* represents the Sparsity Degree, *n* is the total number of questions in the dataset, $N_{i}$denotes the number of distinct words in the *i*-th question, and $|V|$ signifies the size of the vocabulary, which is the total number of unique words across the entire dataset.

This formula provides a nuanced measure of sparsity by not merely counting unique terms but by evaluating their distribution across the dataset. A higher Sparsity Degree indicates a dataset with a broader lexicon and less repetition of individual words, suggesting a rich linguistic diversity. Such diversity is instrumental for developing models capable of understanding and classifying the subtle gradations of subjectivity expressed in questions, as it exposes the model to a wide variety of linguistic constructs and semantic nuances.

When evaluating Sparsity Degree within datasets, it's essential to also consider the number of questions, as high sparsity in larger datasets indicates a challenging achievement of vocabulary diversity, reflecting content richness. This aspect significantly impacts model training, where larger, diverse datasets facilitate learning and generalization, unlike smaller ones where high sparsity may not equate to lexical richness. Thus, assessing both Sparsity Degree and dataset size provides a nuanced perspective, ensuring interpretations are well-founded and reflective of the dataset's true linguistic complexity.

Considering both the Sparsity Degree values and the Question Counts for each dataset (Fig 10), we can derive a more nuanced interpretation of their characteristics and potential value for NLP tasks:

- **FQSD:** With a Sparsity Degree of 0.987 and 10,000 questions, FQSD stands out not only for its exceptionally high vocabulary diversity but also for its substantial size. The combination of a high Sparsity Degree with a large number of questions suggests that FQSD is incredibly rich in unique content, covering a wide range of topics and expressions. This makes it an ideal dataset for developing and testing NLP models, especially for tasks requiring nuanced language understanding, such as FQSC.
- **ConvEx-DS:** Despite having a smaller size of 1,589 questions, ConvEx-DS has a Sparsity Degree of 0.985, indicating a high level of vocabulary diversity. The smaller size combined with high diversity suggests that ConvEx-DS is focused yet varied, potentially offering concentrated examples of specific linguistic phenomena or domain-specific language that could be valuable for targeted NLP applications.
- **Yu et al., 2012:** This dataset is the smallest with only 220 questions, yet it maintains a high sparsity degree of 0.981. The high sparsity degree here could be influenced by the dataset's small size, meaning each question potentially introduces unique terms not seen elsewhere in the dataset. The small size and high sparsity degree could pose challenges for model training due to the limited amount of data.
- **SubjQA:** With 5,192 questions and a Sparsity Degree of 0.983, SubjQA offers a good balance between size and vocabulary diversity. This suggests that SubjQA provides a substantial amount of varied content, making it suitable for training models that need to generalize across a range of topics and styles. Its relatively high Sparsity Degree, combined with a moderate question count, indicates that it is both broad and rich in unique linguistic content.

In summary, FQSD, with its extensive size and the highest sparsity degree, stands out as an ideal dataset for training robust NLP models equipped to handle a broad spectrum of expressions and topics.

**Revised Visualizations for Figures 9 and 10 (revised version):**

**Fig 9.** Visualizing the Root Type-Token Ratio (RTTR) and the Corrected Type-Token Ratio (CTTR) across the FQSD, ConvEx-DS, Yu et al., 2012, and SubjQA datasets, showcasing the Lexical Diversity of each dataset.

**Fig 10.** Visualizing the Sparsity Degree and Total Question Count across the FQSD, ConvEx-DS, Yu et al., 2012, and SubjQA datasets, showcasing the Data Sparsity of each dataset.

**2. Figure Quality and Explanations:**
The figures in the paper need to be improved. They lack informative captions, making it challenging for readers to understand their content. It's crucial to provide clear and concise explanations for each figure, including details on how certain metrics, like lexical count in Figure 8 or the measurement of syntactic and semantic dimensions, were derived. Authors should briefly describe the techniques or methodologies employed to calculate these measures, making it easier for readers to replicate or understand the experiments.

**Response to Reviewer Comment 2: Figure Quality and Explanations**

Dear Reviewer,

Thank you for your constructive feedback regarding the figures captions and Explanations in our manuscript. We acknowledge the importance of informative and clear figure captions and for better understanding and replication of our experiments.

In response to your valuable feedback, we have taken several steps to address these concerns comprehensively:

- **Revised Figure Captions:** Based on your recommendation, all captions have been thoroughly revised to ensure they are not only informative but also succinct, providing clear explanations of the depicted data and the methodologies used for analysis. Below are the added/revised captions:
- **Released Code on GitHub:** Alongside revising our "4.1.2. Comparative Analysis" for clarity, we've provided supplementary code on GitHub (<https://github.com/mahsamb/FSQD/blob/main/Figures.ipynb>), showcasing the use of Matplotlib, Seaborn, pandas, NLTK, and spaCy for figure creation and data processing, offering step-by-step data processing guides, explaining our visualization choices for clarity and accessibility, and sharing the actual Python code for each figure with detailed comments for transparency and reproducibility.
- **Overhauled "4.1.2. Comparative Analysis" Section:** We have almost completely reworked this crucial subsection of "4.1. Dataset Evaluation" to better articulate the rationale behind our choice of metrics for evaluating the dataset depicted in the figures. This revision aims to provide readers with a clearer understanding of the metrics' significance and how they align with our research objectives.

We have updated our manuscript with these revised captions and look forward to any further feedback or suggestions you may have.

**To honor the reviewer's input and ensure the thoroughness of our response, we also include this addition here in our response letter:**

**Below are the newly added or revised captions for the figures in our revised manuscript:**

**Fig 1.** Top 30 TF-IDF Scores of Nouns and Noun Phrases in FQSD.

**Fig 2.** Top 20 TF-IDF Scores of Adverbs/Adjectives in FQSD Categorized by Subjectivity Comparison-Form Classes: a) CO b) CS c) SO d) SS

**Fig 3.** Histograms of TF-IDF Adverbs/Adjectives Scores in FQSD Categorized by Subjectivity Comparison-Form Classes: a) CO b) CS c) SO d) SS

**Fig 4.** Distribution of Total Question Count and Multi-Sentence Question Count across the FQSD, ConvEx-DS, Yu et al., 2012, and SubjQA datasets, showcasing the Size and Structural Analysis of each dataset

**Fig 5.** Distribution of the Total Word Count and Unique Word Count across the FQSD, ConvEx-DS, Yu et al., 2012, and SubjQA datasets, showcasing the Lexical Richness of each dataset.

**Fig 6.** Distribution of the Average Words per Sentence, Average Sentence Length, Average Word Length, and Average Syllables per Word across the FQSD, ConvEx-DS, Yu et al., 2012, and SubjQA datasets, showcasing the Linguistic Complexity of each dataset.

**Fig 7.** Visualizing the Average Parse Tree Depth across the FQSD, ConvEx-DS, Yu et al., 2012, and SubjQA datasets, showcasing the Syntactic Complexity of each dataset.

**Fig 8.** Visualizing the Mean Dependency Distance (MDD) across the FQSD, ConvEx-DS, Yu et al., 2012, and SubjQA datasets, showcasing the Dependency Analysis of each dataset.

**Fig 9.** Visualizing the Root Type-Token Ratio (RTTR) and the Corrected Type-Token Ratio (CTTR) across the FQSD, ConvEx-DS, Yu et al., 2012, and SubjQA datasets, showcasing the Lexical Diversity of each dataset.

**Fig 10.** Visualizing the Sparsity Degree and Total Question Count across the FQSD, ConvEx-DS, Yu et al., 2012, and SubjQA datasets, showcasing the Data Sparsity of each dataset.

**Below are the newly added sections related to code and data availability in our revised manuscript:**

**6. Code and Data Availability**

To ensure the reproducibility of our findings and facilitate further research in this area, the dataset used in this study and the corresponding code have been made publicly available. The dataset can be accessed at https://github.com/mahsamb/FSQD, and the code repository is available at <https://github.com/mahsamb/FSQD/blob/main/Figures.ipynb>.

**Below are the newly revised sections related to our explanations of metrics for evaluating the dataset depicted in the figures in our revised manuscript.**

**4.1.2. Comparative Analysis**

In this section, we undertake a comparative analysis to evaluate the FQSD dataset alongside three existing datasets commonly utilized in Fine-grained subjectivity classification research: the dataset of Yu et al. [1], ConvEx-DS [3], and SubjQA [2]. This analysis aims to provide insights into the unique strengths and comprehensive nature of FQSD, particularly in the context of FQSC. By examining various textual, syntactic, and semantic dimensions, we aim to highlight the distinct attributes and potential utility of FQSD as a gold-standard resource for researchers and practitioners in the field.

**Navigating Datasets for Question Subjectivity**

In this section, different datasets currently utilized in the realm of various tasks of Fine-grained subjectivity classification research are explored. In the following, each dataset's attributes, domains, and how they align or diverge from the objectives of the study are detailed:

Dataset from Yu et al. [1]: It is currently the only one closely related to our study, capable of covering all three tasks considered in this research. It comprises 220 questions from the cell phone domain (Refer to Table 6 for detailed statistics). It is a foundational resource but limited in its scope and complexity. It solely contains single-sentence questions, which may not be a true representation of intricate real-world queries.

ConvEx-DS Dataset [3]: A newer dataset, comprising user question intents in the hotel domain. It contains 1,589 questions that cover both subjectivity and comparison-form classifications (Details in Table 7). It doesn't encompass subjective-type questions and not related to the smartphone domain.

SubjQA Dataset [2]: The SubjQA dataset [2] offers a wealth of subjective and objective questions from six domains (Details in Table 8). While useful, it lacks features crucial to our study: it doesn't include comparison or subjective-type questions and isn't focused on the smartphone domain which is central to our research.

**Benchmarking Against Existing Datasets**

In this section, a comparative analysis is undertaken to position the FQSD dataset against three existing datasets commonly used in the field: the dataset of Yu et al. [1], ConvEx-DS [3], and SubjQA [2]. The aim is to illuminate the unique strengths and comprehensive nature of FQSD, especially in the context of FQSC. The metrics are divided into two main categories: Textual Dimensions and Syntactic and Semantic Dimensions. Each category contains various sub-metrics, offering a multi-faceted understanding of the datasets. By highlighting the distinctive attributes and sheer scale of FQSD, the potential of it as a gold-standard resource for researchers and practitioners alike is emphasized.

**Textual Dimensions**

In this section, the dataset's textual characteristics are explored by analyzing its Size, Lexical Diversity, and Complexity. The dataset's Size and Structural Analysis are assessed through the number of questions it contains and the prevalence of multi-sentence questions, offering insight into its structural depth. Lexical Richness, including the Total and Unique Word Counts, reveal the dataset's vocabulary richness and linguistic variety. Furthermore, Lexical Complexity is examined through metrics like Average Words per Sentence, Average Sentence Length, Average Word Length, and Average Syllables per Word. These analyses, supported by specific metrics and visual aids, provide a comprehensive overview of the dataset's textual landscape, shedding light on its implications for complex language processing tasks and potential applications. This meticulous examination aims to understand the dataset's capacity to support advanced NLP challenges by highlighting its structural intricacies and linguistic diversity.

In the following sections, we will compare these metrics across four different datasets, offering a comparative analysis that highlights the unique characteristics, strengths, and limitations of each dataset concerning the others. This analysis, underpinned by specific metrics and visual aids, provides a detailed snapshot of each dataset's textual makeup and its potential implications.

**Size and Structural Analysis**

Understanding linguistic structures and vocabulary breadth is pivotal for NLP research, directly impacting model effectiveness. The analysis assesses both data volume and the syntactic intricacies within datasets to ensure they offer a diverse range of training examples and pose sufficient linguistic challenges for model refinement. By evaluating the Total Question Count for dataset size and using Multi-Sentence Question Count as a complexity metric—indicative of nuanced information structures—insights into each dataset's quantitative and qualitative dimensions are gained. This comprehensive approach informs dataset selection for NLP development, aiming to enhance models' linguistic understanding and generation capabilities.

To quantify these metrics, we initially segmented the text from each dataset into individual sentences using a regular expression. This process targeted common sentence-ending punctuation—specifically the terminal punctuation markers of '.', '!', and '?'—to delineate sentence boundaries. The Multi-Sentence Count metric was derived by algorithmically identifying sentences within each question based on terminal punctuation markers and calculating the proportion of questions containing more than one such marker.

Fig 4 presents a comparative analysis of datasets by the total number of questions (bar graph) and the count of multi-sentence questions (line graph). It reveals FQSD's dominance in both Total Question Count and Multi-Sentence Question Count, marking it as the most extensive and structurally complex corpus among those studied. While SubjQA ranks second in question volume, its multi-sentence question count is much lower, indicating a simpler structure relative to its size. This compact visualization efficiently highlights the balance between dataset volume and complexity, crucial for selecting optimal corpora for NLP development, with FQSD offering a rich linguistic environment for comprehensive model training.

Fig 4. Distribution of Total Question Count and Multi-Sentence Question Count across the FQSD, ConvEx-DS, Yu et al., 2012, and SubjQA datasets, showcasing the Size and Structural Analysis of each dataset.

**Lexical Richness**

In this investigation, we place a significant emphasis on the lexical composition of NLP datasets to determine their applicability for complex language tasks. Lexical metrics, such as the Total Word Count and Unique Word Count, offer insights into the textual richness and the diversity of the vocabulary. These metrics are crucial for evaluating the potential of datasets to provide varied linguistic input necessary for training robust NLP models [39-41]. By examining the total count of words alongside the count of unique words, the assessment not only evaluates the volume of linguistic content available but also its variety, which is indicative of the potential complexity and nuance that NLP models must grapple with.

To quantify these metrics, each dataset was first segmented into individual sentences. These sentences were then further refined by stripping extraneous whitespace and excluding empty sequences, ensuring a focus on substantive textual content. Subsequently, we applied Python's regular expressions for pattern matching to extract words, treating the boundary between word characters and non-word characters as the delimiter. This approach not only facilitated a comprehensive word count but also ensured that variations in case were normalized, thereby maintaining consistency in the unique word identification process.

Fig 5 reveals the outcomes of this lexical exploration, highlighting the FQSD dataset for its exceptional lexical breadth, as indicated by its high Total Word Count and significant count of unique words. This suggests not only a vast corpus but also a high degree of vocabulary diversity, making it an ideal resource for NLP tasks requiring extensive linguistic variation. In contrast, SubjQA, while smaller in total word volume, also exhibits a noteworthy proportion of unique terms, underscoring its value in providing varied linguistic inputs. This detailed comparison, as presented in Fig 5, serves as a critical resource for selecting datasets that offer the lexical depth necessary for advanced NLP model development and evaluation.

Fig 5. Distribution of the Total Word Count and Unique Word Count across the FQSD, ConvEx-DS, Yu et al., 2012, and SubjQA datasets, showcasing the Lexical Richness of each dataset.

**Lexical Complexity**

In this linguistic analysis, we sought to capture the textual complexity of various datasets by evaluating both the structural and lexical properties of the text. This encompassed a multifaceted approach where we examined Average Words per Sentence [42, 43], Average Sentence Length [42, 44], Average Word Length [45, 47], and Average Syllable Count per Word [42,46,47]. These metrics collectively paint a picture of the textual density and readability, which are indicative of the cognitive load required for comprehension and have implications for the complexity of NLP tasks.

We used the re-library for regular expressions to segment texts into sentences and words. A specialized function was implemented to assess phonetic complexity, systematically estimating the syllable count of each word. This function employs a rule-based algorithm that processes words in lowercase for consistency and uses heuristic methods to tally syllables. It accounts for the phonetic rules of English, such as incrementing the syllable count when encountering vowels at the beginning of a word or following a consonant, and decrementing for silent terminal 'e's, with exceptions like 'le' endings. A final validation step ensures that every word is attributed at least one syllable, adhering to the fundamental structure of language. This meticulous syllable estimation enriches our textual complexity analysis, providing an integral measure that, despite its algorithmic simplicity, yields a robust gauge of the phonetic dimension within the corpora.

Fig 6 presents the results of this analysis in a visually accessible format, with each bar representing a different aspect of textual complexity across the datasets. The FQSD dataset distinctly outperformed in all four assessed metrics: Average Words per Sentence, Average Sentence Length, Average Word Length, and Average Syllables per Word. This comprehensive lead in lexical metrics underscores FQSD's extensive linguistic structure and phonetic complexity. Following in second place, the 'Yu et al., 2012' dataset showed commendable lexical density and complexity, albeit not matching the breadth of FQSD. The 'ConvEx-DS' dataset occupied the third position, indicating a moderate level of textual intricacy. In contrast, 'SubjQA' registered the lowest values across these metrics, suggesting a relative simplicity in its textual composition when compared to the others. This hierarchy of datasets in terms of textual complexity provides pivotal insights for NLP research.

Fig 6. Distribution of the Average Words per Sentence, Average Sentence Length, Average Word Length, and Average Syllables per Word across the FQSD, ConvEx-DS, Yu et al., 2012, and SubjQA datasets, showcasing the Linguistic Complexity of each dataset.

**Syntactic and Semantic Dimensions**

In the "Syntactic and Semantic Dimensions" section, we scrutinize the datasets' structural and linguistic depth using metrics such as Average Parse Tree Depth, Mean Dependency Distance, Root Type-Token Ratio (RTTR), Corrected Type-Token Ratio (CTTR), and Sparsity Degree, complemented by visual references. This exploration reveals the Syntactic Complexity, showcasing the intricacies of sentence structures; Dependency Analysis, highlighting word interconnections; and Lexical Diversity, measuring vocabulary richness. Additionally, we assess Data Sparsity to understand the presence of rare elements, crucial for modeling challenges in NLP tasks. These analyses collectively illuminate the datasets' capabilities and limitations, providing a nuanced understanding of their syntactic and semantic attributes essential for advanced linguistic processing and application development.

**Syntactic Complexity**

Syntactic Complexity refers to the hierarchical structure and arrangement of elements within sentences, serving as a key indicator of the cognitive and linguistic challenges a text presents. In this study, a detailed approach is employed to quantify this complexity through the analysis of Average Parse Tree Depths across four datasets, highlighted in Fig 7. The role of nouns and noun phrases is central to this measure, as they often form the core constituents of the sentence structure, serving as subjects, objects, or complements. The depth of the parse tree reflects the level of nestedness and the complexity of these syntactic constructions. Trees with deeper structures often contain noun phrases with additional modifying elements, such as adjectives and prepositional phrases, which contribute to the intricacy of the language used in the text.

The Average Parse Tree Depths—a result of calculating the longest path from the root to any leaf within the tree—serves as an aggregate indicator of the dataset's syntactic complexity [48-50]. This metric is particularly insightful for understanding the variations and intricacies in the datasets, crucial for the development of robust NLP systems. The presence of complex noun phrases within these trees indicates the potential cognitive load required to process and understand the text, which is a vital consideration in NLP applications like machine translation and information extraction.

The analysis revealed in Fig 7 shows that the FQSD dataset exhibits the highest level of syntactic complexity, implying a dense layering of linguistic constructs within its parse trees. In contrast, the Yu et al., 2012 dataset presents a lower complexity level, suggesting more direct and potentially simpler syntactic structures. The ability to discern these differences is essential when curating corpora for NLP tasks, especially in scenarios where the depth of linguistic analysis, such as identifying subjective nuances within text, is critical.

Fig 7. Visualizing the Average Parse Tree Depth across the FQSD, ConvEx-DS, Yu et al., 2012, and SubjQA datasets, showcasing the Syntactic Complexity of each dataset.

**Dependency Analysis**

In this study, a method is utilized to quantify syntactic complexity across various text datasets by calculating the Mean Dependency Distance (MDD), a metric indicative of linguistic intricacy [51-53]. Utilizing the spaCy library, the MDD metric gauges the average number of words that separate a word from its syntactic head within a sentence. Specifically, the MDD is derived by parsing each sentence to identify these dependencies, calculating the absolute differences between the indices of each word and its head, and then averaging these distances across the entire text. Higher MDD values indicate sentences with greater complexity, where words are, on average, further apart from their syntactic heads, while lower values suggest a tighter, more direct sentence structure.

Fig 8 illustrates the MDD values for four distinct datasets, derived from the application of a spaCy-based NLP model. The FQSD dataset exhibits the highest MDD, signifying its status as the most syntactically complex corpus among those studied. This complexity could pose more challenges but also offers potentially more opportunities for NLP models to learn nuanced language patterns. In contrast, the lower MDD of SubjQA suggests simpler syntax, potentially facilitating comprehension by NLP models. The ConvEx-DS and Yu et al., 2012 datasets have moderate MDD values, denoting a balanced syntactic structure. The differences in MDD demonstrated in Fig 8 highlight the variability in syntactic construction across datasets and emphasize the importance of considering syntactic complexity in NLP model development and dataset selection.

Fig 8. Visualizing the Mean Dependency Distance (MDD) across the FQSD, ConvEx-DS, Yu et al., 2012, and SubjQA datasets, showcasing the Dependency Analysis of each dataset.

**Lexical Diversity**

The metric of lexical diversity, determining the spread of unique words, provides a window into the text's varied linguistic fabric and complexity. The metric of lexical diversity, determining the spread of unique words, provides a window into the text's varied linguistic fabric and complexity [54]. In this study, we utilize the Root Type-Token Ratio (RTTR) and the Corrected Type-Token Ratio (CTTR), leveraging the spaCy NLP library, to analyze and quantify this aspect of textual data. These metrics are essential for evaluating the richness of a dataset's vocabulary, which is an important factor in various NLP applications.

The RTTR [55] is a normalization of the Type-Token Ratio (TTR) [55], which is the number of unique words (types) divided by the total number of words (tokens) in a text. Since TTR is affected by the length of the text, RTTR is used to standardize this measure across texts of different lengths by dividing the number of unique words by the square root of the total number of words, thus providing a more consistent metric of lexical diversity regardless of text size.

The CTTR [55] further corrects for the influence of text length on TTR. It divides the number of unique words by the square root of twice the total number of words, offering a more balanced measure of lexical richness, particularly in longer texts, where the sheer volume can skew the traditional TTR.

Fig 9 assesses the lexical diversity of four datasets through the RTTR and the CTTR, with RTTR values depicted in dark blue and CTTR in light blue. The FQSD dataset is distinguished by the highest RTTR and CTTR values, indicating substantial lexical diversity suitable for robust NLP training. In contrast, Yu et al., 2012 exhibit the least diversity, with the lowest RTTR and CTTR values, pointing to a more limited or focused lexical scope. SubjQA and ConvEx-DS display moderate diversity, suggesting a balanced range of vocabulary. This visualization underscores the varying lexical complexities across datasets, which is essential for selecting appropriate corpora for specific NLP tasks.

Fig 9. Visualizing the Root Type-Token Ratio (RTTR) and the Corrected Type-Token Ratio (CTTR) across the FQSD, ConvEx-DS, Yu et al., 2012, and SubjQA datasets, showcasing the Lexical Diversity of each dataset.

**Data Sparsity**

In the pursuit of enhancing our understanding and analysis of datasets used for FQSC, we adopted a metric for assessing the linguistic diversity of each dataset: Sparsity Degree. This metric is pivotal in quantifying the extent of vocabulary diversity, which is essential for tasks requiring nuanced language interpretation, such as subjectivity classification. The Sparsity Degree is calculated using the formula [56]:

$$Sd=1- \frac{1}{n} \sum_{i=1}^{n} \frac{N_{i}}{\left| V \right|} (1)$$

Where *Sd* represents the Sparsity Degree, *n* is the total number of questions in the dataset, $N_{i}$denotes the number of distinct words in the *i*-th question, and $|V|$ signifies the size of the vocabulary, which is the total number of unique words across the entire dataset.

This formula provides a nuanced measure of sparsity by not merely counting unique terms but by evaluating their distribution across the dataset. A higher Sparsity Degree indicates a dataset with a broader lexicon and less repetition of individual words, suggesting a rich linguistic diversity. Such diversity is instrumental for developing models capable of understanding and classifying the subtle gradations of subjectivity expressed in questions, as it exposes the model to a wide variety of linguistic constructs and semantic nuances.

When evaluating Sparsity Degree within datasets, it's essential to also consider the number of questions, as high sparsity in larger datasets indicates a challenging achievement of vocabulary diversity, reflecting content richness. This aspect significantly impacts model training, where larger, diverse datasets facilitate learning and generalization, unlike smaller ones where high sparsity may not equate to lexical richness. Thus, assessing both Sparsity Degree and dataset size provides a nuanced perspective, ensuring interpretations are well-founded and reflective of the dataset's true linguistic complexity.

Considering both the Sparsity Degree values and the Question Counts for each dataset (Fig 10), we can derive a more nuanced interpretation of their characteristics and potential value for NLP tasks:

- **FQSD:** With a Sparsity Degree of 0.987 and 10,000 questions, FQSD stands out not only for its exceptionally high vocabulary diversity but also for its substantial size. The combination of a high Sparsity Degree with a large number of questions suggests that FQSD is incredibly rich in unique content, covering a wide range of topics and expressions. This makes it an ideal dataset for developing and testing NLP models, especially for tasks requiring nuanced language understanding, such as FQSC.
- **ConvEx-DS:** Despite having a smaller size of 1,589 questions, ConvEx-DS has a Sparsity Degree of 0.985, indicating a high level of vocabulary diversity. The smaller size combined with high diversity suggests that ConvEx-DS is focused yet varied, potentially offering concentrated examples of specific linguistic phenomena or domain-specific language that could be valuable for targeted NLP applications.
- **Yu et al., 2012:** This dataset is the smallest with only 220 questions, yet it maintains a high sparsity degree of 0.981. The high sparsity degree here could be influenced by the dataset's small size, meaning each question potentially introduces unique terms not seen elsewhere in the dataset. The small size and high sparsity degree could pose challenges for model training due to the limited amount of data.
- **SubjQA:** With 5,192 questions and a Sparsity Degree of 0.983, SubjQA offers a good balance between size and vocabulary diversity. This suggests that SubjQA provides a substantial amount of varied content, making it suitable for training models that need to generalize across a range of topics and styles. Its relatively high Sparsity Degree, combined with a moderate question count, indicates that it is both broad and rich in unique linguistic content.

In summary, FQSD, with its extensive size and the highest sparsity degree, stands out as an ideal dataset for training robust NLP models equipped to handle a broad spectrum of expressions and topics.

**Fig 10.** Visualizing the Sparsity Degree and Total Question Count across the FQSD, ConvEx-DS, Yu et al., 2012, and SubjQA datasets, showcasing the Data Sparsity of each dataset.

3. Dataset Schema:
Including the schema of the dataset in a JSON or similar format would be beneficial. This would provide readers with a clearer understanding of the data used in the experiments and help them better interpret the results.

**Response to Reviewer Comment 3: Dataset Schema:**

Dear Reviewer,

Thank you for your valuable suggestion to include the schema of our dataset in a JSON format. We agree that providing a detailed schema will enhance the clarity and comprehensibility of the data used in our experiments.

In response to your recommendation, we have developed JSON schemas for our dataset, referred to as FQSD, as well as for the three additional Yu et al. [1], SubjQA [2], and ConvEx-DS [3], that our study compares. These schemas are intended to furnish a detailed account of the data structure utilized in our experiments, thus enhancing the readability and interpretability of our results for the audience.

For FQSD, our primary dataset, the schema outlines columns such as "Question", "Label_FSQD", "Label_Subjectivity", "Label_ComparisonForm", "Label_Subjectivity_ComparisonForm", and "Label_SubjectivityType". This dataset is intricately crafted to support a variety of fine-grained question subjectivity classification tasks, including FSQD, Subjectivity, Comparison Form, Subjectivity_ComparisonForm, and SubjectivityType.

Yu et al. [1], mirrors FQSD in terms of structure, containing the same 6 columns, which facilitates a direct comparison across different datasets while maintaining a consistent framework for our analysis.

SubjQA [2], however, is more focused, containing only the "Question" and "Label_Subjectivity" columns. This specificity aligns with the dataset's targeted approach towards understanding subjectivity in isolation.

ConvEx-DS [3] includes "Question", "Label_Subjectivity", "Label_ComparisonForm", "Label_Subjectivity_ComparisonForm", providing a middle ground in terms of complexity and focus, allowing for nuanced analyses of subjectivity and comparison forms within questions.

By presenting these schemas in a JSON format, we aim to offer a comprehensive overview of each dataset's structure, emphasizing the significance of each column and the classification tasks they support. These detailed schemas for Yu et al. [1], SubjQA [2], and ConvEx-DS [3] ensure a transparent and thorough understanding of the data structures employed in our comparative analysis.

These schemas have now been included as supplementary material in our submission (https://github.com/mahsamb/FSQD/blob/main/FSQD-Json-dataset.zip), and the code related to visualizing the dataset is at <https://github.com/mahsamb/FSQD/blob/main/Figures.ipynb>. We believe this enhancement will significantly aid readers and researchers in accurately interpreting the results of our experiments and in facilitating the replication of our study as needed.

Thank you for guiding us to enhance our manuscript. We look forward to any further feedback you may have.

4. Explanation of LIME Evaluations:
a) The paper should offer an intuitive explanation of LIME (Local Interpretable Model-agnostic Explanations) evaluations. This will help readers comprehend the significance and implications of using LIME in the context of QA NLP work.

**Response to Reviewer's Comment on LIME Evaluations:**

In response to the valuable feedback regarding the inclusion of an intuitive explanation of LIME (Local Interpretable Model-agnostic Explanations) evaluations, I would like to express my sincere appreciation for this insightful suggestion. Understanding the importance of transparency and interpretability in QA NLP work, we have now incorporated a comprehensive section dedicated to elucidating the role and significance of LIME within our study. To specifically address the reviewer's suggestion, we have revised the related section, now titled "4.2.2. LIME Visualizations: Unveiling Decision Dynamics on Unseen Data," which delves into the practical application and impact of LIME evaluations in our research.

**To honor the reviewer's input and ensure the thoroughness of our response, we also include this addition here in our response letter:**

**4.2.2.	LIME Visualizations: Unveiling Decision Dynamics on Unseen Data**

To elucidate the decision-making processes of the model and ensure the interpretability of its predictions, Local Interpretable Model-agnostic Explanations (LIME) was employed. LIME provides a transparent view into the model's reasoning, highlighting the words and phrases that significantly influence its predictions. In Fig 11, LIME visualizations of word influence in the model's predictions for Instances 1 and 2 on the Yu et al. [1] dataset are effectively illustrated.

On the left side of each figure, the prediction probabilities for different classes are displayed. "Other" represents the cumulative probability of six additional classes due to space limitations. Despite "Other" showing a higher cumulative probability, the individual probabilities for "TSS" in Fig 11 (a) and "YSS" in Fig 11 (b) stand out, marking them as the model's top predictions for the respective instances. Specifically, in sub-figure (a), although "Other" encapsulates a cumulative probability of 0.51 for RCS, ACS, YCS, YSS, and NCO classes, "TSS" demonstrates the highest individual class probability at 0.23. This makes "TSS" the model's leading prediction for the question "What features of iPhone 3gs are disliked by reviewers?

The right side of each figure visualizes the word-level contributions to the model's prediction. Words that positively influence the prediction towards "TSS" or "YSS" extend the bars to the right. Conversely, words that negatively influence these predictions extend to the left. For instance, in Fig 11 (a), the LIME analysis reveals that words such as "What" and "disliked" carry significant weight, with "disliked" being especially impactful, suggesting a strong subjective sentiment. Similarly, in Fig 11 (b), words like "fragile" and "broken" significantly influence the model's classification of the sentence as "YSS", indicating subjectivity based on personal experience or opinion.

The section titled "Text with highlighted words" at the bottom presents the actual questions with key influential words highlighted. This provides a direct correlation to the visualized word weights and allows for an intuitive understanding of the model's decision-making process.

Overall, these LIME visualizations are instrumental in dissecting the RoBERTa model's reasoning on a per-word basis. They offer deep insights into the model's logic, ensuring that the predictions are based on relevant textual evidence.

**5. Benchmark Choice:**
a) The authors should provide a clear rationale for choosing RoBERTa as the benchmark model. Explaining why RoBERTa was selected over other models can help readers understand the motivations behind this choice and the implications for the study**.**

**Response to Reviewer's Comment on Rationale for Choosing RoBERTa:**

Dear Reviewer,

Thank you for your constructive feedback regarding our selection of RoBERTa as the benchmark model for our study. We appreciate the opportunity to clarify our decision and the evaluation metrics employed.

**Rationale for Choosing RoBERTa:**

- **Performance and Advancements:** RoBERTa (Robustly Optimized BERT Pretraining Approach) represents an advancement over the original BERT model, offering improved performance on several natural language processing (NLP) benchmarks. This superior performance is primarily due to RoBERTa's optimized training process, which includes longer training with more data, bigger batches, and removal of the Next Sentence Prediction objective used in BERT.
- **Exceptional Architecture:** RoBERTa surpasses BERT and XLNet with its dynamic masking technique and removal of the NSP (Next Sentence Prediction) task during pre-training, enriching its understanding of context and nuances in language for complex NLP tasks.
- **Alignment with Study Objectives:** Our study required a model capable of understanding complex language structures and nuances, particularly in the context of question-answering systems. RoBERTa's enhanced pretraining approach made it an ideal candidate, as it demonstrates a better understanding of context and sentence relationships, which are critical for our analysis.
- **Benchmarking Against Other Models:** Prior to finalizing RoBERTa, we conducted preliminary experiments with several models, including the original BERT and XLNet. Our findings showed that RoBERTa outperformed these models in key areas relevant to our research, thereby solidifying our decision to use it as the benchmark model.
- **Superiority in Cross-Domain Applications:** Demonstrating unparalleled performance across diverse datasets like FQSD, ConvEx-DS, and SubjQA, RoBERTa's robust adaptability and consistent accuracy highlight its preeminence in fine-grained question subjectivity classification.
- **Evaluation Metrics for Question Classification:** For our specific task of question classification, we focused on metrics that evaluate the model's accuracy in categorizing questions correctly. These included standard classification metrics such as accuracy, precision, recall, and F1-score, which are more suitable for our research objectives.
- **Insightful Interpretability:** RoBERTa exhibits excellent decision-making clarity across diverse datasets, as revealed by LIME analysis

**To honor the reviewer's input and ensure the thoroughness of our response, we also include this addition here in our response letter:**

**3.3. Choice of Model Architecture**

In the exploration of FQSC, RoBERTa emerged as the primary model of choice, a decision anchored in its exceptional architectural sophistication and pre-training advancements. Distinctively, RoBERTa's approach to pre-training—marked by an expansive dataset and iterative process augmented with dynamically changing masking patterns—catapults its linguistic comprehension to levels well-suited for the discerning differentiation between objective and subjective questions, alongside the precise categorization of question forms. The dynamic masking feature of RoBERTa signifies a critical innovation within the broader spectrum of context-aware language models, distinguishing it from other approaches in this category, such as static masking and permutation-based strategies. This feature is instrumental in broadening the model's contextual understanding, thereby amplifying its generalization capabilities to adeptly navigate the subtleties of unseen questions.

Crucially, RoBERTa abandons the Next-Sentence Prediction (NSP) task—a move that empirical evidence suggests significantly strengthens its efficacy in downstream applications, including the intricate domain of question classification. The model's deep Transformer layers, foundational to its architecture, serve as a robust feature extraction mechanism that can be particularly useful for the inherent complexities of question classification tasks. This architectural depth, combined with RoBERTa's proven adaptability across varied linguistic contexts and classification paradigms, underscores its optimal fit for our research goals. The model's demonstrated excellence across multiple NLP benchmarks further solidifies our confidence in its selection, ensuring our methodology is underpinned by the most advanced NLP technology available. Moreover, RoBERTa's capacity for granular text analysis is essential for delineating the ten subjectivity classes our study investigates. This careful selection of RoBERTa, based on superior performance and architectural merits, helps our study to be at the forefront of advancing FQSC.

Building on this foundation, the RoBERTa-base model, specifically chosen for our task, consists of 12 transformer layers, 768 hidden units per layer, and a total of 125 million parameters. This architecture is designed to provide a deep and nuanced understanding of language, which is critical for the complex requirements of FQSC. By combining RoBERTa with LIME, we enhance not only the transparency of our analytical process but also enrich the understanding of the model's decision-making framework.

**4.4.2. Cross-Dataset Performance Evaluation of RoBERTa, BERT, and XLNet in Fine-Grained Subjectivity Tasks**

In this section, the effectiveness of prominent Transformer architectures in the specialized task of FQSC was meticulously evaluated. This comparative analysis included RoBERTa, BERT, and XLNet, which were assessed on Precision, Recall, and F-score metrics. These metrics, averaged over five independent runs, were derived using a 5-fold stratified cross-validation technique to confirm the robustness and generalizability of the evaluation.

Tables 14, 15, and 16 provide a granular view of the performance of Transformer models RoBERTa, BERT, and XLNet across different tasks and datasets. Table 13 focuses on the FQSC task within the FQSD dataset, showcasing the models' precision, recall, and F-score over five independent runs. Table 14 evaluates these models on the subjectivity-comparison form classification task in the ConvEx-DS dataset, again over five runs, highlighting their robustness and consistency. Lastly, Table 15 examines the models' performance on the subjectivity classification task in the SubjQA dataset, detailing domain-specific precision, recall, and F-scores to underline their adaptability and effectiveness across diverse contexts.

In Table 16 and 17, experiments conducted on the SubjQA Dataset were performed with a learning rate (lr) of 3e-5, in contrast to the learning rate of 1e-5 used in other tables, notably Table 11. This deviation was strategically chosen due to the unique characteristics of the SubjQA dataset, where BERT and XLNet exhibited tendencies to overfit. To ensure a fair comparison across models, the same lr of 3e-5 was adopted for RoBERTa when evaluating this particular dataset, despite RoBERTa not showing the same overfitting issues. This adjustment underscores our commitment to maintaining equitable experimental conditions and accurately assessing model performance across diverse datasets.

Table 17 clearly outlines the performance metrics for each model across a variety of datasets, namely SubjQA, ConvEx-DS, and FQSD. The tasks range from general subjectivity classification to the more nuanced subjectivity-comparison form classification. The learning rate (lr) for each model is noted, revealing the tailored optimization strategies for each dataset. In light of the varying performances of BERT and XLNet—each outperforming the other in different contexts—it's RoBERTa that consistently demonstrates superiority and steadiness across all evaluated tasks and datasets. This observation highlights RoBERTa's robustness and its capability to maintain high performance levels, making it a standout model for fine-grained question subjectivity classification and related tasks.

RoBERTa's architectural sophistication and comprehensive training regime are central to its preeminence. The dynamic masking feature is a significant enhancement over BERT's static masking and XLNet's permutation-based learning, providing RoBERTa with unmatched contextual flexibility. This innovation is pivotal in enabling RoBERTa to capture the fine distinctions of subjectivity within questions across all datasets examined.

The focused approach of RoBERTa on the masked language model task, to the exclusion of other tasks like NSP, directly correlates with its elevated performance levels. The extensive and varied corpus on which RoBERTa was trained has endowed the model with an intricate understanding of language, evident in its exceptional performance on datasets like FQSD.

In conclusion, this thorough analysis reasserts RoBERTa's role as the definitive model for FQSC. Its consistent top-tier performance, advanced architectural features, and targeted training approach highlight its unmatched ability to discern nuanced subjectivity within questions, evidencing its practical utility and adaptability to diverse datasets.

**Table 14.** Comparative Analysis of Transformer Models' Performance (LR=1e-5) on the FQSC Task Across the FQSD Dataset Over Five Independent Runs

| **Model** | **K-fold** | **Precision** | **Recall** | **F-Score** |
| --- | --- | --- | --- | --- |
| **Roberta** | **1** | 96.8 | 96.6 | 96.6 |
|  | **2** | 96.0 | 95.9 | 95.8 |
|  | **3** | 96.9 | 96.8 | 96.8 |
|  | **4** | 97.7 | 97.7 | 97.6 |
|  | **5** | 97.8 | 97.8 | 97.8 |
|  | **Average** | **97.04** | **97.16** | **97.12** |
| **Bert** | **1** | 96.7 | 96.6 | 96.6 |
|  | **2** | 95.4 | 95.4 | 95.4 |
|  | **3** | 97.5 | 97.5 | 97.5 |
|  | **4** | 96.4 | 96.3 | 96.2 |
|  | **5** | 96.5 | 96.5 | 96.5 |
|  | **Average** | **96.50** | **96.46** | **96.44** |
| **XLnet** | **1** | 97.1 | 97.0 | 97.0 |
|  | **2** | 97.0 | 97.1 | 97.0 |
|  | **3** | 97.4 | 97.4 | 97.4 |
|  | **4** | 96.2 | 96.2 | 96.2 |
|  | **5** | 97.0 | 96.8 | 96.9 |
|  | **Average** | **96.94** | **96.91** | **96.90** |

**Table 15.** Comparative Analysis of Transformer Models' Performance (LR=1e-5) on the Subjectivity-Comparison Form Classification Task Across the ConvEx-DS Dataset [3] Over Five Independent Runs

| **Model** | **K-fold** | **Precision** | **Recall** | **F-Score** |
| --- | --- | --- | --- | --- |
| Roberta | 1 | 89.23 | 90.10 | 89.66 |
|  | 2 | 91.42 | 89.25 | 90.32 |
|  | 3 | 90.33 | 89.37 | 89.85 |
|  | 4 | 90.44 | 91.26 | 90.84 |
|  | 5 | 91.18 | 90.21 | 90.69 |
|  | Average | **90.52** | **90.04** | **90.07** |
| Bert | 1 | 83.02 | 81.76 | 81.85 |
|  | 2 | 83.15 | 83.64 | 83.09 |
|  | 3 | 84.46 | 84.59 | 84.44 |
|  | 4 | 79.25 | 80.18 | 78.75 |
|  | 5 | 85.45 | 84.54 | 84.90 |
|  | Average | **83.86** | **82.94** | **82.60** |
| XLnet | 1 | 80.92 | 81.44 | 80.54 |
|  | 2 | 82.90 | 82.07 | 80.30 |
|  | 3 | 81.00 | 81.44 | 79.18 |
|  | 4 | 78.64 | 78.61 | 77.90 |
|  | 5 | 84.86 | 85.80 | 85.11 |
|  | Average | **81.864** | **81.872** | **80.206** |

**Table 16. Comparative Analysis of Transformer Models' Performance (LR=3e-5) on the Subjectivity Classification Task Across the SubjQA Dataset [2] Over Five Independent Runs**

| **Model** | **Domain** | **Precision** | **Recall** | **F-Score** |
| --- | --- | --- | --- | --- |
| Roberta | Books | 89.69 | 89.95 | 89.79 |
|  | Electronic | 92.46 | 92.92 | 92.54 |
|  | Grocery | 88.39 | 86.51 | 87.09 |
|  | Movies | 94.65 | 94.70 | 94.66 |
|  | TripAdvisor | 93.36 | 93.15 | 93.23 |
|  | Restaurants | 92.48 | 92.02 | 92.20 |
|  | Average | **91.27** | **91.90** | **91.98** |
| XLnet | Books | 86.26 | 86.12 | 86.19 |
|  | Electronic | 89.64 | 90.70 | 89.69 |
|  | Grocery | 79.87 | 79.53 | 79.69 |
|  | Movies | 82.96 | 82.01 | 78.51 |
|  | TripAdvisor | 89.14 | 88.58 | 88.80 |
|  | Restaurants | 93.79 | 89.57 | 90.55 |
|  | Average | **86.27** | **86.63** | **86.23** |
| Bert | Books | 90.44 | 87.08 | 87.96 |
|  | Electronic | 92.01 | 89.38 | 90.24 |
|  | Grocery | 85.06 | 85.58 | 85.26 |
|  | Movies | 92.71 | 92.59 | 92.26 |
|  | TripAdvisor | 91.06 | 91.32 | 91.12 |
|  | Restaurants | 92.93 | 91.41 | 91.87 |
|  | Average | **90.99** | **89.60** | **89.51** |

Table 17. Comparative Analysis of Transformer Models' Performance on Fine-Grained Subjectivity Tasks Across Multiple Datasets Over Five Independent Runs

| **Domain** | **Task** | **LR** | **Model** | **Precision** | **Recall** | **F-Score** |
| --- | --- | --- | --- | --- | --- | --- |
| SubjQA [2] | Subjectivity Classification | 3e-5 | Roberta | 91.27 | 91.90 | 91.98 |
|  |  |  | XLnet | 86.27 | 86.63 | 86.23 |
|  |  |  | Bert | 90.99 | 89.60 | 89.51 |
| ConvEx-DS [3] | Subjectivity-ComparisonForm  Classification | 1e-5 | Roberta | 90.52 | 90.04 | 90.07 |
|  |  |  | XLnet | 81.86 | 81.87 | 80.21 |
|  |  |  | Bert | 83.86 | 82.94 | 82.60 |
| FQSD | Fine-grained Question Subjectivity Classification (FQSC) | 1e-5 | Roberta | 97.04 | 97.16 | 97.12 |
|  |  |  | XLnet | 96.94 | 96.91 | 96.90 |
|  |  |  | Bert | 96.50 | 96.46 | 96.44 |

We hope this response adequately addresses your queries about our choice of RoBERTa and the evaluation approach used in our study. We believe that these methodological choices strengthen the validity of our research findings.

Thank you once again for your valuable feedback, and we look forward to any further suggestions or inquiries.

b) Additionally, it's advisable to reference related works like <https://doi.org/10.1186/s12859-022-04751-6> in the literature that discuss the use of QA and NLP evaluation.

Dear Reviewer,

We are grateful for the recommendation to reference the article <https://doi.org/10.1186/s12859-022-04751-6>, which discusses the utilization of QA and NLP evaluation in depth. Following this suggestion, we have incorporated this reference into Section "2.2. Question Classification: Historical to Fine-grained Perspectives" of our article.

**To honor the reviewer's input and ensure the thoroughness of our response, we also highlight this addition here in our response letter:**

While the above-mentioned studies primarily focus on the product domain, recent research has extended into new areas. The creation and utilization of the CoQUAD datasets [32] for COVID-19 question-answering showcase a practical application of fine-grained question classification in a healthcare context. This work highlights the ability of ASQA systems to adapt and provide accurate, domain-specific information, addressing the complex challenges of classifying questions within specific domains like epidemiology, public health, and clinical care. This expansion reflects the evolving trends in ASQA research towards developing nuanced and effective question understanding mechanisms across diverse fields.

c) Additionally, it would be valuable to mention whether the QA systems were evaluated using exact match metrics or similarity metrics based on BERT or other models.

Dear Reviewer,

Thank you for your comment regarding the evaluation of QA systems. We would like to clarify that our study focuses on question classification within the realm of Natural Language Processing (NLP), rather than on providing answers in a QA system. As such, the evaluation metrics typically used for assessing answer accuracy in QA systems, such as exact match metrics or similarity metrics based on BERT or other models, are not directly applicable to our research.

Instead, our evaluation is centered on the classification accuracy of questions. We have employed the following metrics suited for classification tasks:

- **Precision and Recall:** These metrics evaluate the model's performance in terms of its ability to correctly identify questions of a specific category (precision) and its capability to identify all relevant instances of that category (recall).
- **F1-Score:** We also use the F1-score, a harmonic mean of precision and recall, which provides a balance between these two metrics, especially valuable in scenarios of class imbalance.

We believe these metrics appropriately reflect the objectives of our study in question classification and offer a comprehensive assessment of our model's performance.

Thank you for the opportunity to clarify the focus of our study and the evaluation metrics employed. We appreciate your valuable feedback and guidance.

**6. Data Quality Section:**
a) While the paper briefly touches on data quality concerns, it would be beneficial to have a dedicated section that thoroughly addresses data quality issues. This can include discussions any potential biases in the dataset or annotators biases and how it is dealth. Providing this information will enhance the transparency and credibility of the research.

Dear Reviewer,

Thank you for your insightful feedback regarding the need for a dedicated section on data quality and the enhancement of figure quality and captions in our manuscript. We recognize the importance of these aspects in strengthening the transparency and credibility of our research.

In response to your suggestions, we have made the following revisions to our manuscript:

In response to your suggestions, in addition to the previously detailed sections "Unique Features of the FQSD" and "3.2.3. Annotation Process and Inter-Annotator Agreement," we have made the following revisions to our manuscript:

- **Data Quality Section:** We have added a new section titled "3.2. Data Quality and Integrity," which thoroughly addresses data quality concerns. This section discusses the meticulous dataset preparation, annotation processes, and the implementation of bias mitigation strategies to ensure the FQSD dataset's integrity and applicability in NLP research. We believe this addition significantly enhances the understanding of how data quality was maintained and validated throughout our research.

**To honor the reviewer's input and ensure the thoroughness of our response, we also include this addition here in our response letter:**

**3.2. Data Quality and Integrity**

Ensuring the standards of data quality and integrity is foundational to the development and utility of the FQSD dataset. This segment elaborates on the processes employed to cultivate a dataset that meets the demands of NLP research. Through comprehensive dataset preparation, the integration of unique features, a carefully designed annotation process, and stringent measures to gauge inter-annotator agreement, we establish an approach that prioritizes precision, reliability, and ethical considerations in dataset creation.

**3.2.1. Dataset Preparation and Specifications**

In this section, an exhaustive and detailed account of the rigorous preparation and underlying specifications that form the cornerstone of this dataset's development is provided. This includes an in-depth exploration of the methods employed, the criteria applied in the selection and adaptation of data, and the strategic considerations that guided the compilation process to ensure the dataset's integrity and relevance to its intended research applications.

- **Dataset Inspiration and Collection:** Our initial dataset drew inspiration from user-generated content on question-answering platforms such as Quora and Amazon, specifically in the smartphone domain. However, sourcing questions that met the specific criteria proved challenging. The criteria necessitated the explicit mention of compared entities' names, clarity, and specificity in phrasing, and alignment with our predefined categories.
- **Adaptation and Creation of the FQSD Dataset:** There is limited availability of questions directly fitting our research needs. To fill the gaps, we adapted existing content and created new questions, ensuring a rich variety of data. This included:
  - **Modify Sentences:** Transforming existing sentences into questions suitable for our study.
  - **Content Adjustment:** Adding, removing, or altering sections of the questions to enhance specificity and relevance.
  - **Creating Fictional Data:** Supplementing the dataset with fictional but plausible questions to cover a broader range of scenarios and inquiries.
- **Validation and Approval Process:** The altered and newly created questions were subjected to a rigorous validation process, overseen by expert commentators knowledgeable in writing and conceptual aspects within the smartphone domain. Their approval ensured the dataset's relevance and adherence to our research objectives.
- **Bias Mitigation Strategies in Dataset Compilation:** To ensure the FQSD dataset minimizes biases inherent in question selection and framing, several strategies tailored to question classification tasks were implemented. Firstly, the dataset was compiled from a wide range of online platforms beyond mainstream sites like Quora and Amazon, including niche forums and industry-specific Q&A sites. This approach ensures a variety of question types and topics, reducing the risk of selection bias towards popular or commonly discussed issues. Secondly, during the adaptation and creation of questions, we carefully balanced the dataset to include questions from different smartphone categories, reflecting a broad spectrum of user intents and information needs. This diversity helps in minimizing content bias, ensuring our dataset supports the development of classification models capable of understanding a wide range of question formats and types. Finally, to address annotator bias in question classification, annotators were provided with extensive guidelines designed to maintain consistency and neutrality in question interpretation and classification. Regular calibration sessions were held to align annotators’ understanding and ensure a uniform standard was applied across the dataset.
- **Compliance and Ethical Considerations:** Our data collection, modification, and creation processes were conducted in strict compliance with the terms and conditions of the source platforms. Ethical considerations were paramount, including the exclusion of any personally identifiable information and ensuring the privacy and confidentiality of individuals associated with the initial data sources.

b) Please proof read the paper again and bring quality figures with captions and how these are made (used some techniques like for visual?)

**Response to Reviewer Comment 6: Proofreading, Figures, and Captions Enhancement:**

**Proofreading Enhancement**: In response to your valuable feedback, we have undertaken a thorough proofreading of our manuscript to ensure clarity, coherence, and correctness in our presentation. This comprehensive review focused on addressing grammatical errors, refining the narrative for better readability, and verifying the accuracy of our technical descriptions and citations. We appreciate this suggestion, as it underscores the importance of maintaining high standards of written communication in academic publishing. We believe that these efforts have significantly improved the manuscript's quality, making our research findings and contributions clearer and more accessible to readers.

**Enhancement of Figures and Captions:** We have reviewed all figures in the manuscript to ensure they are of high quality and effectively convey the intended information. Each figure now has a detailed caption explaining its content, significance, and the methodologies or techniques used for its creation. This enhancement aims to improve the clarity and informativeness of the visual elements of our paper. While we have successfully enhanced the quality of all figures, we faced technical limitations with improving the LIME visualization for RoBERTa's predictions (figure 11) due to its inherent presentation in HTML format. Despite exploring various methods, converting this particular visualization into a high-quality image format, akin to outputs from libraries like Matplotlib, proved to be challenging. Consequently, we were forced to resort to taking a screenshot to include this critical visualization in our manuscript, ensuring the detail and clarity essential for understanding the model's decision dynamics were preserved.

We hope that these revisions address your concerns and improve the quality of our submission. We appreciate your guidance in this process and look forward to any further feedback you may have.

**To honor the reviewer's input and ensure the thoroughness of our response, we also include this addition here in our response letter:**

**Below are the newly added or revised captions for the figures in our manuscript:**

**Fig 1.** Top 30 TF-IDF Scores of Nouns and Noun Phrases in FQSD.

**Fig 2.** Top 20 TF-IDF Scores of Adverbs/Adjectives in FQSD Categorized by Subjectivity Comparison-Form Classes: a) CO b) CS c) SO d) SS

**Fig 3.** Histograms of TF-IDF Adverbs/Adjectives Scores in FQSD Categorized by Subjectivity Comparison-Form Classes: a) CO b) CS c) SO d) SS

**Fig 4.** Distribution of Total Question Count and Multi-Sentence Question Count across the FQSD, ConvEx-DS, Yu et al., 2012, and SubjQA datasets, showcasing the Size and Structural Analysis of each dataset

**Fig 5.** Distribution of the Total Word Count and Unique Word Count across the FQSD, ConvEx-DS, Yu et al., 2012, and SubjQA datasets, showcasing the Lexical Richness of each dataset.

**Fig 6.** Distribution of the Average Words per Sentence, Average Sentence Length, Average Word Length, and Average Syllables per Word across the FQSD, ConvEx-DS, Yu et al., 2012, and SubjQA datasets, showcasing the Linguistic Complexity of each dataset.

**Fig 7.** Visualizing the Average Parse Tree Depth across the FQSD, ConvEx-DS, Yu et al., 2012, and SubjQA datasets, showcasing the Syntactic Complexity of each dataset.

**Fig 8.** Visualizing the Mean Dependency Distance (MDD) across the FQSD, ConvEx-DS, Yu et al., 2012, and SubjQA datasets, showcasing the Dependency Analysis of each dataset.

**Fig 9.** Visualizing the Root Type-Token Ratio (RTTR) and the Corrected Type-Token Ratio (CTTR) across the FQSD, ConvEx-DS, Yu et al., 2012, and SubjQA datasets, showcasing the Lexical Diversity of each dataset.

**Fig 10.** Visualizing the Sparsity Degree and Total Question Count across the FQSD, ConvEx-DS, Yu et al., 2012, and SubjQA datasets, showcasing the Data Sparsity of each dataset.

**Response to Reviewer Comment 6: How Figures Were Made:**

Thank you for emphasizing the critical importance of ensuring that our visualizations effectively convey the intended message. We appreciate this constructive feedback as it highlights an essential aspect of research communication.

In this regard, we have undertaken two main tasks to address your concerns:

- **Overhauled "4.1.2. Comparative Analysis" Section:** We have almost completely reworked this crucial subsection of "4.1. Dataset Evaluation" to better articulate the rationale behind our choice of metrics for evaluating the dataset depicted in the figures. This revision aims to provide readers with a clearer understanding of the metrics' significance and how they align with our research objectives.
- **Released Code on GitHub:** In addition to the detailed process outlined for creating our figures, "4.1.2. Comparative Analysis" of our article, that were almost completely revised to ensure coherence and clarity, we have also provided the code as supplementary material on GitHub at <https://github.com/mahsamb/FSQD/blob/main/Figures.ipynb>. This repository includes comprehensive documentation of the libraries, methods, and visualization techniques employed in our study. Here, we illustrate:
- **Libraries Used:** We detail our use of data visualization libraries such as Matplotlib and Seaborn for generating figures, pandas for data manipulation, and both NLTK and spaCy for natural language processing. These tools were pivotal in analyzing and visualizing the rich textual data within our dataset.
- **Methods Applied:** Step-by-step guides on how we processed our data, including the deployment of Textual Dimensions for content analysis and Syntactic and Semantic Dimensions for evaluating the complexity and meaning within our datasets. These methodologies were integral to our article, providing a deeper understanding of the datasets used.
- **Visualization Techniques:** We provide an explanation of the specific visualization types and color schemes tailored for accessibility. This includes illustrating histograms, scatter plots, and bar graphs to best represent our data, along with adopting color-blind friendly palettes to ensure inclusivity.
- **Code for Each Figure:** The actual Python code used to generate figures is shared, accompanied by comments explaining each step. This transparency ensures that our methods are not just reproducible but also understandable, allowing for the replication of our visualizations or the adaptation of our methods for other studies.

By providing this supplementary code repository, we aim to not only enhance the transparency of our research process but also to facilitate the reproducibility of our results. This initiative is driven by our commitment to open science, enabling interested researchers to replicate our visualizations or adapt our methods for their own studies, thus contributing to the broader scientific community.

**To honor the reviewer's input and ensure the thoroughness of our response, we also include this addition here in our response letter:**

**4.1.2. Comparative Analysis**

In this section, we undertake a comparative analysis to evaluate the FQSD dataset alongside three existing datasets commonly utilized in Fine-grained subjectivity classification research: the dataset of Yu et al. [1], ConvEx-DS [3], and SubjQA [2]. This analysis aims to provide insights into the unique strengths and comprehensive nature of FQSD, particularly in the context of FQSC. By examining various textual, syntactic, and semantic dimensions, we aim to highlight the distinct attributes and potential utility of FQSD as a gold-standard resource for researchers and practitioners in the field.

**Navigating Datasets for Question Subjectivity**

In this section, different datasets currently utilized in the realm of various tasks of Fine-grained subjectivity classification research are explored. In the following, each dataset's attributes, domains, and how they align or diverge from the objectives of the study are detailed:

Dataset from Yu et al. [1]: It is currently the only one closely related to our study, capable of covering all three tasks considered in this research. It comprises 220 questions from the cell phone domain (Refer to Table 6 for detailed statistics). It is a foundational resource but limited in its scope and complexity. It solely contains single-sentence questions, which may not be a true representation of intricate real-world queries.

ConvEx-DS Dataset [3]: A newer dataset, comprising user question intents in the hotel domain. It contains 1,589 questions that cover both subjectivity and comparison-form classifications (Details in Table 7). It doesn't encompass subjective-type questions and not related to the smartphone domain.

SubjQA Dataset [2]: The SubjQA dataset [2] offers a wealth of subjective and objective questions from six domains (Details in Table 8). While useful, it lacks features crucial to our study: it doesn't include comparison or subjective-type questions and isn't focused on the smartphone domain which is central to our research.

**Benchmarking Against Existing Datasets**

In this section, a comparative analysis is undertaken to position the FQSD dataset against three existing datasets commonly used in the field: the dataset of Yu et al. [1], ConvEx-DS [3], and SubjQA [2]. The aim is to illuminate the unique strengths and comprehensive nature of FQSD, especially in the context of FQSC. The metrics are divided into two main categories: Textual Dimensions and Syntactic and Semantic Dimensions. Each category contains various sub-metrics, offering a multi-faceted understanding of the datasets. By highlighting the distinctive attributes and sheer scale of FQSD, the potential of it as a gold-standard resource for researchers and practitioners alike is emphasized.

**Textual Dimensions**

In this section, the dataset's textual characteristics are explored by analyzing its Size, Lexical Diversity, and Complexity. The dataset's Size and Structural Analysis are assessed through the number of questions it contains and the prevalence of multi-sentence questions, offering insight into its structural depth. Lexical Richness, including the Total and Unique Word Counts, reveal the dataset's vocabulary richness and linguistic variety. Furthermore, Lexical Complexity is examined through metrics like Average Words per Sentence, Average Sentence Length, Average Word Length, and Average Syllables per Word. These analyses, supported by specific metrics and visual aids, provide a comprehensive overview of the dataset's textual landscape, shedding light on its implications for complex language processing tasks and potential applications. This meticulous examination aims to understand the dataset's capacity to support advanced NLP challenges by highlighting its structural intricacies and linguistic diversity.

In the following sections, we will compare these metrics across four different datasets, offering a comparative analysis that highlights the unique characteristics, strengths, and limitations of each dataset concerning the others. This analysis, underpinned by specific metrics and visual aids, provides a detailed snapshot of each dataset's textual makeup and its potential implications.

**Size and Structural Analysis**

Understanding linguistic structures and vocabulary breadth is pivotal for NLP research, directly impacting model effectiveness. The analysis assesses both data volume and the syntactic intricacies within datasets to ensure they offer a diverse range of training examples and pose sufficient linguistic challenges for model refinement. By evaluating the Total Question Count for dataset size and using Multi-Sentence Question Count as a complexity metric—indicative of nuanced information structures—insights into each dataset's quantitative and qualitative dimensions are gained. This comprehensive approach informs dataset selection for NLP development, aiming to enhance models' linguistic understanding and generation capabilities.

To quantify these metrics, we initially segmented the text from each dataset into individual sentences using a regular expression. This process targeted common sentence-ending punctuation—specifically the terminal punctuation markers of '.', '!', and '?'—to delineate sentence boundaries. The Multi-Sentence Count metric was derived by algorithmically identifying sentences within each question based on terminal punctuation markers and calculating the proportion of questions containing more than one such marker.

Fig 4 presents a comparative analysis of datasets by the total number of questions (bar graph) and the count of multi-sentence questions (line graph). It reveals FQSD's dominance in both Total Question Count and Multi-Sentence Question Count, marking it as the most extensive and structurally complex corpus among those studied. While SubjQA ranks second in question volume, its multi-sentence question count is much lower, indicating a simpler structure relative to its size. This compact visualization efficiently highlights the balance between dataset volume and complexity, crucial for selecting optimal corpora for NLP development, with FQSD offering a rich linguistic environment for comprehensive model training.

Fig 4. Distribution of Total Question Count and Multi-Sentence Question Count across the FQSD, ConvEx-DS, Yu et al., 2012, and SubjQA datasets, showcasing the Size and Structural Analysis of each dataset.

**Lexical Richness**

In this investigation, we place a significant emphasis on the lexical composition of NLP datasets to determine their applicability for complex language tasks. Lexical metrics, such as the Total Word Count and Unique Word Count, offer insights into the textual richness and the diversity of the vocabulary. These metrics are crucial for evaluating the potential of datasets to provide varied linguistic input necessary for training robust NLP models [39-41]. By examining the total count of words alongside the count of unique words, the assessment not only evaluates the volume of linguistic content available but also its variety, which is indicative of the potential complexity and nuance that NLP models must grapple with.

To quantify these metrics, each dataset was first segmented into individual sentences. These sentences were then further refined by stripping extraneous whitespace and excluding empty sequences, ensuring a focus on substantive textual content. Subsequently, we applied Python's regular expressions for pattern matching to extract words, treating the boundary between word characters and non-word characters as the delimiter. This approach not only facilitated a comprehensive word count but also ensured that variations in case were normalized, thereby maintaining consistency in the unique word identification process.

Fig 5 reveals the outcomes of this lexical exploration, highlighting the FQSD dataset for its exceptional lexical breadth, as indicated by its high Total Word Count and significant count of unique words. This suggests not only a vast corpus but also a high degree of vocabulary diversity, making it an ideal resource for NLP tasks requiring extensive linguistic variation. In contrast, SubjQA, while smaller in total word volume, also exhibits a noteworthy proportion of unique terms, underscoring its value in providing varied linguistic inputs. This detailed comparison, as presented in Fig 5, serves as a critical resource for selecting datasets that offer the lexical depth necessary for advanced NLP model development and evaluation.

Fig 5. Distribution of the Total Word Count and Unique Word Count across the FQSD, ConvEx-DS, Yu et al., 2012, and SubjQA datasets, showcasing the Lexical Richness of each dataset.

**Lexical Complexity**

In this linguistic analysis, we sought to capture the textual complexity of various datasets by evaluating both the structural and lexical properties of the text. This encompassed a multifaceted approach where we examined Average Words per Sentence [42, 43], Average Sentence Length [42, 44], Average Word Length [45, 47], and Average Syllable Count per Word [42,46,47]. These metrics collectively paint a picture of the textual density and readability, which are indicative of the cognitive load required for comprehension and have implications for the complexity of NLP tasks.

We used the re-library for regular expressions to segment texts into sentences and words. A specialized function was implemented to assess phonetic complexity, systematically estimating the syllable count of each word. This function employs a rule-based algorithm that processes words in lowercase for consistency and uses heuristic methods to tally syllables. It accounts for the phonetic rules of English, such as incrementing the syllable count when encountering vowels at the beginning of a word or following a consonant, and decrementing for silent terminal 'e's, with exceptions like 'le' endings. A final validation step ensures that every word is attributed at least one syllable, adhering to the fundamental structure of language. This meticulous syllable estimation enriches our textual complexity analysis, providing an integral measure that, despite its algorithmic simplicity, yields a robust gauge of the phonetic dimension within the corpora.

Fig 6 presents the results of this analysis in a visually accessible format, with each bar representing a different aspect of textual complexity across the datasets. The FQSD dataset distinctly outperformed in all four assessed metrics: Average Words per Sentence, Average Sentence Length, Average Word Length, and Average Syllables per Word. This comprehensive lead in lexical metrics underscores FQSD's extensive linguistic structure and phonetic complexity. Following in second place, the 'Yu et al., 2012' dataset showed commendable lexical density and complexity, albeit not matching the breadth of FQSD. The 'ConvEx-DS' dataset occupied the third position, indicating a moderate level of textual intricacy. In contrast, 'SubjQA' registered the lowest values across these metrics, suggesting a relative simplicity in its textual composition when compared to the others. This hierarchy of datasets in terms of textual complexity provides pivotal insights for NLP research.

Fig 6. Distribution of the Average Words per Sentence, Average Sentence Length, Average Word Length, and Average Syllables per Word across the FQSD, ConvEx-DS, Yu et al., 2012, and SubjQA datasets, showcasing the Linguistic Complexity of each dataset.

**Syntactic and Semantic Dimensions**

In the "Syntactic and Semantic Dimensions" section, we scrutinize the datasets' structural and linguistic depth using metrics such as Average Parse Tree Depth, Mean Dependency Distance, Root Type-Token Ratio (RTTR), Corrected Type-Token Ratio (CTTR), and Sparsity Degree, complemented by visual references. This exploration reveals the Syntactic Complexity, showcasing the intricacies of sentence structures; Dependency Analysis, highlighting word interconnections; and Lexical Diversity, measuring vocabulary richness. Additionally, we assess Data Sparsity to understand the presence of rare elements, crucial for modeling challenges in NLP tasks. These analyses collectively illuminate the datasets' capabilities and limitations, providing a nuanced understanding of their syntactic and semantic attributes essential for advanced linguistic processing and application development.

**Syntactic Complexity**

Syntactic Complexity refers to the hierarchical structure and arrangement of elements within sentences, serving as a key indicator of the cognitive and linguistic challenges a text presents. In this study, a detailed approach is employed to quantify this complexity through the analysis of Average Parse Tree Depths across four datasets, highlighted in Fig 7. The role of nouns and noun phrases is central to this measure, as they often form the core constituents of the sentence structure, serving as subjects, objects, or complements. The depth of the parse tree reflects the level of nestedness and the complexity of these syntactic constructions. Trees with deeper structures often contain noun phrases with additional modifying elements, such as adjectives and prepositional phrases, which contribute to the intricacy of the language used in the text.

The Average Parse Tree Depths—a result of calculating the longest path from the root to any leaf within the tree—serves as an aggregate indicator of the dataset's syntactic complexity [48-50]. This metric is particularly insightful for understanding the variations and intricacies in the datasets, crucial for the development of robust NLP systems. The presence of complex noun phrases within these trees indicates the potential cognitive load required to process and understand the text, which is a vital consideration in NLP applications like machine translation and information extraction.

The analysis revealed in Fig 7 shows that the FQSD dataset exhibits the highest level of syntactic complexity, implying a dense layering of linguistic constructs within its parse trees. In contrast, the Yu et al., 2012 dataset presents a lower complexity level, suggesting more direct and potentially simpler syntactic structures. The ability to discern these differences is essential when curating corpora for NLP tasks, especially in scenarios where the depth of linguistic analysis, such as identifying subjective nuances within text, is critical.

Fig 7. Visualizing the Average Parse Tree Depth across the FQSD, ConvEx-DS, Yu et al., 2012, and SubjQA datasets, showcasing the Syntactic Complexity of each dataset.

**Dependency Analysis**

In this study, a method is utilized to quantify syntactic complexity across various text datasets by calculating the Mean Dependency Distance (MDD), a metric indicative of linguistic intricacy [51-53]. Utilizing the spaCy library, the MDD metric gauges the average number of words that separate a word from its syntactic head within a sentence. Specifically, the MDD is derived by parsing each sentence to identify these dependencies, calculating the absolute differences between the indices of each word and its head, and then averaging these distances across the entire text. Higher MDD values indicate sentences with greater complexity, where words are, on average, further apart from their syntactic heads, while lower values suggest a tighter, more direct sentence structure.

Fig 8 illustrates the MDD values for four distinct datasets, derived from the application of a spaCy-based NLP model. The FQSD dataset exhibits the highest MDD, signifying its status as the most syntactically complex corpus among those studied. This complexity could pose more challenges but also offers potentially more opportunities for NLP models to learn nuanced language patterns. In contrast, the lower MDD of SubjQA suggests simpler syntax, potentially facilitating comprehension by NLP models. The ConvEx-DS and Yu et al., 2012 datasets have moderate MDD values, denoting a balanced syntactic structure. The differences in MDD demonstrated in Fig 8 highlight the variability in syntactic construction across datasets and emphasize the importance of considering syntactic complexity in NLP model development and dataset selection.

Fig 8. Visualizing the Mean Dependency Distance (MDD) across the FQSD, ConvEx-DS, Yu et al., 2012, and SubjQA datasets, showcasing the Dependency Analysis of each dataset.

**Lexical Diversity**

The metric of lexical diversity, determining the spread of unique words, provides a window into the text's varied linguistic fabric and complexity. The metric of lexical diversity, determining the spread of unique words, provides a window into the text's varied linguistic fabric and complexity [54]. In this study, we utilize the Root Type-Token Ratio (RTTR) and the Corrected Type-Token Ratio (CTTR), leveraging the spaCy NLP library, to analyze and quantify this aspect of textual data. These metrics are essential for evaluating the richness of a dataset's vocabulary, which is an important factor in various NLP applications.

The RTTR [55] is a normalization of the Type-Token Ratio (TTR) [55], which is the number of unique words (types) divided by the total number of words (tokens) in a text. Since TTR is affected by the length of the text, RTTR is used to standardize this measure across texts of different lengths by dividing the number of unique words by the square root of the total number of words, thus providing a more consistent metric of lexical diversity regardless of text size.

The CTTR [55] further corrects for the influence of text length on TTR. It divides the number of unique words by the square root of twice the total number of words, offering a more balanced measure of lexical richness, particularly in longer texts, where the sheer volume can skew the traditional TTR.

Fig 9 assesses the lexical diversity of four datasets through the RTTR and the CTTR, with RTTR values depicted in dark blue and CTTR in light blue. The FQSD dataset is distinguished by the highest RTTR and CTTR values, indicating substantial lexical diversity suitable for robust NLP training. In contrast, Yu et al., 2012 exhibit the least diversity, with the lowest RTTR and CTTR values, pointing to a more limited or focused lexical scope. SubjQA and ConvEx-DS display moderate diversity, suggesting a balanced range of vocabulary. This visualization underscores the varying lexical complexities across datasets, which is essential for selecting appropriate corpora for specific NLP tasks.

Fig 9. Visualizing the Root Type-Token Ratio (RTTR) and the Corrected Type-Token Ratio (CTTR) across the FQSD, ConvEx-DS, Yu et al., 2012, and SubjQA datasets, showcasing the Lexical Diversity of each dataset.

**Data Sparsity**

In the pursuit of enhancing our understanding and analysis of datasets used for FQSC, we adopted a metric for assessing the linguistic diversity of each dataset: Sparsity Degree. This metric is pivotal in quantifying the extent of vocabulary diversity, which is essential for tasks requiring nuanced language interpretation, such as subjectivity classification. The Sparsity Degree is calculated using the formula [56]:

$$Sd=1- \frac{1}{n} \sum_{i=1}^{n} \frac{N_{i}}{\left| V \right|} (1)$$

Where *Sd* represents the Sparsity Degree, *n* is the total number of questions in the dataset, $N_{i}$denotes the number of distinct words in the *i*-th question, and $|V|$ signifies the size of the vocabulary, which is the total number of unique words across the entire dataset.

This formula provides a nuanced measure of sparsity by not merely counting unique terms but by evaluating their distribution across the dataset. A higher Sparsity Degree indicates a dataset with a broader lexicon and less repetition of individual words, suggesting a rich linguistic diversity. Such diversity is instrumental for developing models capable of understanding and classifying the subtle gradations of subjectivity expressed in questions, as it exposes the model to a wide variety of linguistic constructs and semantic nuances.

When evaluating Sparsity Degree within datasets, it's essential to also consider the number of questions, as high sparsity in larger datasets indicates a challenging achievement of vocabulary diversity, reflecting content richness. This aspect significantly impacts model training, where larger, diverse datasets facilitate learning and generalization, unlike smaller ones where high sparsity may not equate to lexical richness. Thus, assessing both Sparsity Degree and dataset size provides a nuanced perspective, ensuring interpretations are well-founded and reflective of the dataset's true linguistic complexity.

Considering both the Sparsity Degree values and the Question Counts for each dataset (Fig 10), we can derive a more nuanced interpretation of their characteristics and potential value for NLP tasks:

- **FQSD:** With a Sparsity Degree of 0.987 and 10,000 questions, FQSD stands out not only for its exceptionally high vocabulary diversity but also for its substantial size. The combination of a high Sparsity Degree with a large number of questions suggests that FQSD is incredibly rich in unique content, covering a wide range of topics and expressions. This makes it an ideal dataset for developing and testing NLP models, especially for tasks requiring nuanced language understanding, such as FQSC.
- **ConvEx-DS:** Despite having a smaller size of 1,589 questions, ConvEx-DS has a Sparsity Degree of 0.985, indicating a high level of vocabulary diversity. The smaller size combined with high diversity suggests that ConvEx-DS is focused yet varied, potentially offering concentrated examples of specific linguistic phenomena or domain-specific language that could be valuable for targeted NLP applications.
- **Yu et al., 2012:** This dataset is the smallest with only 220 questions, yet it maintains a high sparsity degree of 0.981. The high sparsity degree here could be influenced by the dataset's small size, meaning each question potentially introduces unique terms not seen elsewhere in the dataset. The small size and high sparsity degree could pose challenges for model training due to the limited amount of data.
- **SubjQA:** With 5,192 questions and a Sparsity Degree of 0.983, SubjQA offers a good balance between size and vocabulary diversity. This suggests that SubjQA provides a substantial amount of varied content, making it suitable for training models that need to generalize across a range of topics and styles. Its relatively high Sparsity Degree, combined with a moderate question count, indicates that it is both broad and rich in unique linguistic content.

In summary, FQSD, with its extensive size and the highest sparsity degree, stands out as an ideal dataset for training robust NLP models equipped to handle a broad spectrum of expressions and topics.

**Fig 10.** Visualizing the Sparsity Degree and Total Question Count across the FQSD, ConvEx-DS, Yu et al., 2012, and SubjQA datasets, showcasing the Data Sparsity of each dataset.

We hope that these revisions address your concerns and improve the quality of our submission. We appreciate your guidance in this process and look forward to any further feedback you may have.

6. PLOS authors have the option to publish the peer review history of their article ([what does this mean?](https://journals.plos.org/plosone/s/editorial-and-peer-review-process#loc-peer-review-history)). If published, this will include your full peer review and any attached files.


If you choose “no”, your identity will remain anonymous but your review may still be made public.


**Do you want your identity to be public for this peer review?** For information about this choice, including consent withdrawal, please see our [Privacy Policy](https://www.plos.org/privacy-policy).

Reviewer #1: **Yes:** Shaina Raza

Dear Editor,

I would like to express my appreciation for Reviewer #1, Shaina Raza, for choosing to disclose their identity in the peer review process of our manuscript. This gesture of transparency is highly valued and contributes significantly to the openness and constructive nature of the peer review system.

Reviewer Raza's willingness to be identified speaks to the integrity and confidence in their review, which we found to be both insightful and instrumental in enhancing the quality of our work. The detailed and constructive feedback provided by Reviewer Raza has been greatly beneficial in guiding the revisions to our manuscript.

We believe that such transparency in the peer review process fosters a more collaborative and trustful academic environment, encouraging honest and open communication. We are grateful for the opportunity to have our work reviewed by experts who are committed to upholding these principles.

Thank you once again to Reviewer Raza for their valuable contributions, and to PLOS ONE for upholding a publication process that supports transparency and openness.

[NOTE: If reviewer comments were submitted as an attachment file, they will be attached to this email and accessible via the submission site. Please log into your account, locate the manuscript record, and check for the action link "View Attachments". If this link does not appear, there are no attachment files.]

While revising your submission, please upload your figure files to the Preflight Analysis and Conversion Engine (PACE) digital diagnostic tool, <https://pacev2.apexcovantage.com/>. PACE helps ensure that figures meet PLOS requirements. To use PACE, you must first register as a user. Registration is free. Then, login and navigate to the UPLOAD tab, where you will find detailed instructions on how to use the tool. If you encounter any issues or have any questions when using PACE, please email PLOS at [figures@plos.org](mailto:figures@plos.org). Please note that Supporting Information files do not need this step.

**Conclusion:**

In conclusion, we have carefully considered each of your suggestions and have made comprehensive revisions to our manuscript to address these points. We believe these changes significantly enhance the clarity, depth, and overall quality of our work. Thank you once again for your constructive feedback, which has been invaluable in strengthening our contribution to the field.

We look forward to your further comments and hope that our revisions meet your expectations.

Sincerely, Dr. Afsaneh Fatemi

*In compliance with data protection regulations, you may request that we remove your personal registration details at any time.* [*(Remove my information/details)*](https://www.editorialmanager.com/pone/login.asp?a=r)*. Please contact the publication office if you have any questions.*
